# Novel dual-function near-infrared II fluorescence and PET probe for tumor delineation and image-guided surgery†
†Electronic supplementary information (ESI) available. See DOI: 10.1039/c7sc04774f


**DOI:** 10.1039/c7sc04774f

**Published:** 2018-01-08

**Authors:** Yao Sun, Xiaodong Zeng, Yuling Xiao, Changhao Liu, Hua Zhu, Hui Zhou, Ziyang Chen, Fuchun Xu, Jule Wang, Mengyue Zhu, Junzhu Wu, Mei Tian, Hong Zhang, Zixin Deng, Zhen Cheng, Xuechuan Hong

**Affiliations:** a State Key Laboratory of Virology , Key Laboratory of Combinatorial Biosynthesis and Drug Discovery (MOE) and Hubei Province Engineering and Technology Research Center for Fluorinated Pharmaceuticals , Wuhan University School of Pharmaceutical Sciences , Wuhan 430071 , China . Email: xhy78@whu.edu.cn; b Molecular Imaging Program at Stanford (MIPS) , Bio-X Program , Department of Radiology , Stanford University , CA 94305 , USA . Email: zcheng@stanford.edu; c Key Laboratory of Pesticides and Chemical Biology , Ministry of Education , College of Chemistry , Central China Normal University , Wuhan 430079 , China; d Medical College , Tibet University , Lasa , 850000 , China; e Hubei Provincial Key Laboratory of Developmentally Originated Disease , Center for Experimental Basic Medical Education , Wuhan University , Wuhan 430071 , China; f Department of Nuclear Medicine , The Second Hospital of Zhejiang University School of Medicine , 88 Jiefang Road , Hangzhou , 310009 , China . Email: meitian@zju.edu.cn

## Abstract

Accurate tumor identification is essential in cancer management. Incomplete excision of tumor tissue, however, negatively affects the prognosis of the patient. To accomplish radical excision of tumor tissue, radiotracers can be used that target tumor tissue and can be detected using a gamma probe during surgery. Intraoperative fluorescence imaging could allow accurate real-time tumor delineation. Herein, a novel dual-modal imaging platform using base-catalyzed double addition of thiols into a propiolamide scaffold has been developed, allowing for the highly efficient and selective assembly of various thiol units in a protecting-group-free manner. The first small-molecule based αvβ_3_-targeted NIR-II/PET probe **^68^Ga-SCH2** was concisely generated *via* this strategy and subsequently evaluated in mice bearing the U87MG xenograft. Excellent imaging properties such as good tumor uptake, high tumor contrast and specificity, tumor delineation and image-guided surgery were achieved in the small animal models. These attractive results of **^68^Ga-SCH2** allow it to be a promising αvβ_3_-targeted NIR-II/PET probe for clinical translation.

## Abstract 2

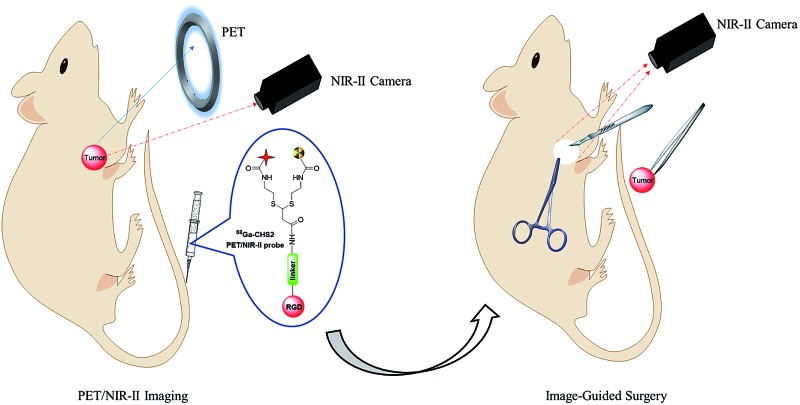
The first small-molecule based αvβ_3_-targeted NIR-II/PET dual-modal probes *via* base-catalyzed thiol-addition chemistry were concisely assembled and evaluated.

## Introduction

Maximizing tumor excision and minimizing collateral damage are the primary goals of cancer surgery. Incomplete excision of tumor tissue, however, negatively affects the prognosis of the patient. Evidence from numerous experimental and clinical studies has demonstrated the significant benefits of molecular imaging in targeted surgery with preoperative molecular diagnostic screening, fluorescence image-guided surgery and postoperative imaging.^1^–^5^ So far only two small molecules, methylene blue and indocyanine green (ICG), have been approved by the FDA, emitting within the traditional NIR-I region (750–900 nm) and with the desirable features of rapid renal excretion and low toxicity.^6^,^7^ Despite their imaging qualities being superior to those of visible wavelengths in accurate real-time tumor delineation, photostability and the penetration depth of emitted light in biological tissues remain challenging. To address this limitation, fluorophores emitting within the second near-infrared region (NIR-II, 1000–1700 nm) show great promise. NIR-II probes possess high spatial resolution and deep-tissue penetration due to reduced photon scattering and diminished auto-fluorescence.^8^–^13^ At present, several classes of fluorescent NIR-II probes including small molecules,^14^–^17^ carbon nanotubes,^18^,^19^ Ag_2_S dots^20^,^21^ and polymers^22^ have been actively employed for NIR-II fluorescence for superior vascular imaging, cerebral imaging, lymphatic imaging and imaging-guided surgery.^23^,^24^

A surge in dual-modal instrumentation development for image-guided surgery and other clinical applications has sparked the discovery of more dual-modal contrast agents to clearly delineate the localization and expression of biochemical markers, and effectively track the tumor with high-resolution and high-sensitivity.^2^,^4^–^6^,^25^–^37^ Using dual-modality probes for sentinel lymph node mapping and image-guided surgery shows promising results in prostate cancer,^28^,^35^ breast cancer^36^ and head and neck cancer.^37^ Although radio-guided surgery and intraoperative fluorescence imaging are powerful techniques to improve accurate tumor detection and resection, they have their limitations. Specific radiotracers can be used that target tumor tissue and that can be detected using a gamma probe during surgery. Yet this technique cannot provide a precise delineation of the tumor and resection margins.^25^–^37^ The addition of a NIR-I fluorescent label could help to overcome this limitation. Intraoperative NIR-I fluorescence imaging could allow accurate real-time tumor delineation, but the penetration depth of emitted light in biological tissue is limited. There is no doubt that developing novel NIR-II dual-modal molecular probes for *in vivo* multimodal imaging applications thus has high significance and direct impact on the field of biomedicine. Although a variety of small-molecule based scaffolds have been successfully explored to construct NIR-I dual-modal imaging probes, widespread applications of these probes in molecular imaging and biomedicine are heavily hampered due to multiple-step synthetic challenges, tedious protection–deprotection strategies and low chemo-selectivity.^28^,^38^,^39^ Therefore, developing effective molecular platforms for the construction of dual-modal NIR-II probes with high chemo-selectivity is urgently demanded.

**Fig. 1:**
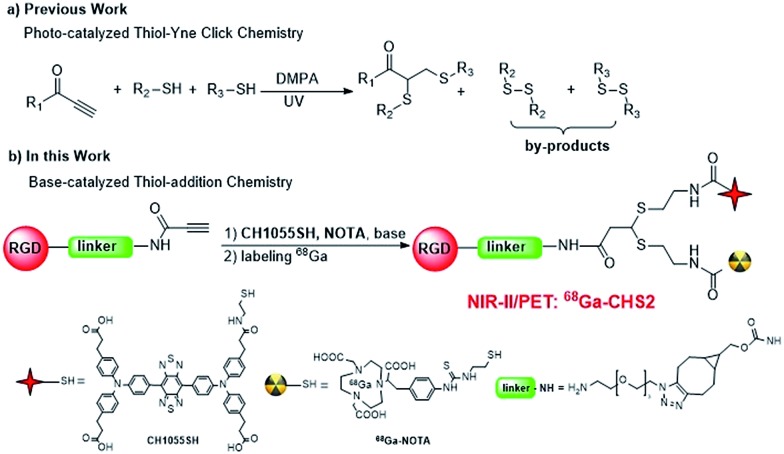
A new strategy for the construction of NIR-II dual-modal imaging probes. (a) The previous photo-catalyzed thiol-yne reaction; (b) the approach discussed in the present work.

In a proof-of-concept study, a NIR-II and PET imaging dual-modality probe **^68^Ga-CHS2** (Fig. 1 and S1†) for the first time has been concisely prepared *via* novel base-catalyzed thiol-yne chemistry for tumor delineation and image-guided surgery. Two thiolated units **CH1055** and ^68^Ga-1,4,7-triazacyclononane-triacetic acid (NOTA) are controlled and integrated across a simple linear terminal alkyne such as propiolamide (PA) sequentially by base-induction. A variety of light-sensitive molecules (fluorescent probes and proteins) can be assembled straightforwardly and by-products such as disulfides can be minimized significantly.^39^ In this paper, a highly innovative method to improve the surgical resection of U87MG tumors *in vivo* has been demonstrated using an integrin αvβ_3_ targeted dual-label tracer that can be detected with a PET probe, and then with a fluorescence NIR-II camera during surgery.

## Results and discussion

Firstly, cysteamine (2-aminoethanethiol, CA or 2-AET) and benzylamine modified PA scaffolds **1** were chosen for optimization studies (Table 1). Interestingly, the mono-thiol-addition reaction was carried out spontaneously in moderate yield with high regio-selectivity (**1a**, entry 1, Fig. S1 and S2†). However, the yields of mono-adducts (**1a**) were increased to 70–83% when DIPEA, NEt_3_ and DBU (0.10 equiv.) were applied (entries 3–5). The *cis* : *trans* ratios and isolated yields of **1a** varied slightly in a variety of solvents and different carbon lengths of thiol substrates (C6 and C10) in the presence of DIPEA (entries 6–9, Fig. S5†). Interestingly, the yield of **1a** was significantly reduced to 5% when cysteamine hydrochloride was introduced instead of cysteamine (Table S1†). Adding the radical scavenger TEMPO (10 equiv.) slightly reduced the yield of adducts suggesting a non-radical addition mechanism (entry 2). Therefore, a base-catalyzed thiol-addition mechanism is tentatively proposed (Fig. S3†).^40^ The double-adduct **1b** could be obtained solely in 67% yield in the presence of 1,5,7-triazabicyclo[4.4.0]dec-5-ene (TBD, 0.20 equiv.) with excess thiols for an extended period of time (entry 10, Fig. S4 and Table S2†). The homo-dimer product *N*-benzyl-3,3-bis(benzylthio)propenamide (**1e**) or hetero-dimer adduct 3-((10-aminodecyl)thio)-3-((2-aminoethyl)thio)-*N*-benzylpropanamide (**1f**) can be controllably constructed by the double addition of benzyl mercaptan, or the subsequent addition of cysteamine and 10-aminodecane-1-thiol in 78% and 63% yields respectively (Fig. 1 and S6–S9†). The *cis* and *trans* structures of mono-adducts and homo and heterodimer-adducts were all confirmed by ESI-MS and ^1^H/^13^C NMR analyses (see ESI†).

**Table 1:** Base-catalyzed thiol-addition chemistry

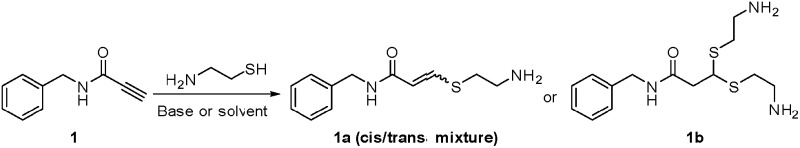						
Entry^*a*^	Catalyst	*t* [min]	Thiol (equiv.)	Solvent	Yield^*b*^ (%)	Product
1	Spontaneous	30	1.0 eq.	DMF	52	**1a** (*cis* : *trans* = 90 : 10)
2	TEMPO (10 equiv.)	30	1.0 eq.	DMF	49	**1a** (*cis* : *trans* = 90 : 10)
3	NEt_3_ (0.1 equiv.)	30	1.0 eq.	DMF	70	**1a** (*cis* : *trans* = 83 : 17)
4	DIPEA (0.1 equiv.)	30	1.0 eq.	DMF	80	**1a** (*cis* : *trans* = 90 : 10)
5	DBU (0.1 equiv.)	30	1.0 eq.	DMF	83	**1a** (*cis* : *trans* = 80 : 20)
6	DIPEA (0.1 equiv.)	30	1.0 eq.	DCM	82	**1a** (*cis* : *trans* = 87 : 13)
7	DIPEA (0.1 equiv.)	30	1.0 eq.	MeOH	85	**1a** (*cis* : *trans* = 85 : 15)
8	DIPEA (0.1 equiv.)	30	1.0 eq.	THF	80	**1a** (*cis* : *trans* = 85 : 15)
9	DIPEA (0.1 equiv.)	30	1.0 eq.	PBS/DMF (8.2)	80	**1a** (*cis* : *trans* = 87 : 13)
10	TBD (0.2 equiv.)	60	4.0 eq.	DMF	67	**1b**

^*a*^Reaction conditions: [alkyne] = 0.25 M in solvents, catalyst = 0.1 or 0.2 equiv.

^*b*^Isolated yield calculated on the basis of the starting alkyne and referred to the major products.

Further studies focused on the investigation of this methodology for the construction of molecular imaging probes. A variety of peptide-functional scaffolds [c(RGDfK)-PA (**2a**), AE105-PA (**2b**) and JMV594-PA (**2c**)] and thiol units [2-AET-NOTA (**Y1**), 2-AET-Cy5.5 (**Y2**) and 2-AET-CH1055 (**Y3**)] were prepared respectively (see ESI†). As shown in Table 2, single modal integrin αvβ_3_-targeted PET (**3a**), NIR-I (**3b**) and NIR-II (**3c**) probes have been successfully generated with 58%, 60% and 60% yields respectively (entries 1–3). In order to expand the library of peptide-based NIR-II probes, novel AE105 and JMV594 peptide-based NIR-II probes (**3d** and **3e**) were synthesized in 61% and 60% yields respectively (entries 4–5). Furthermore, treatment of the AE105-PA scaffold with 2-AET (4 equiv.) resulted in the formation of a double-adduct (**4a**) in 58% yield (entry 6). Finally, an integrin αvβ_3_-targeted NIR-I/PET probe (**4b**) was successfully prepared in 47% yield (entry 7).

**Table 2:** Base-catalyzed addition of various functional molecules into propiolamide-based scaffolds

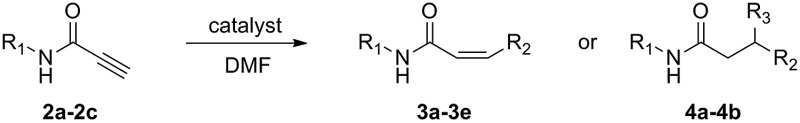					
Entry^*a*^	PA scaffolds^*b*^	Thiol^*c*^	*t* [h]	Product	Yield (%)
1	R_1_ = c(RGDfK) (**2a**)	2-AET-NOTA (**Y1**)	1.5	R_2_ = 2-AET-NOTA (**3a**)	58
2	R_1_ = c(RGDfK) (**2a**)	2-AET-Cy5.5 (**Y2**)	1.5	R_2_ = 2-AET-Cy5.5 (**3b**)	60
3	R_1_ = c(RGDfK) (**2a**)	2-AET-CH1055 (**Y3**)	1.5	R_2_ = 2-AET-CH1055 (**3c**)	60
4	R_1_ = AE105 (**2b**)	2-AET-CH1055 (**Y3**)	1.5	R_2_ = 2-AET-CH1055 (**3d**)	61
5	R_1_ = JMV594 (**2c**)	2-AET-CH1055 (**Y3**)	1.5	R_2_ = 2-AET-CH1055 (**3e**)	60
6	R_1_ = AE105 (**2b**)	2-AET	2	R_2_ = R_3_ = 2-AET (**4a**)	58
7	R_1_ = c(RGDfK) (**2a**)	2-AET-NOTA (**Y1**), 2-AET-Cy5.5 (**Y2**)	2	R_2_ = 2-AET-NOTA, R_3_ = 2AET-Cy5.5 (**4b**)	47

^*a*^The catalyst DIPEA for entries 1–5; TBD for entries 6–7.

^*b*^c(RGDfK) = cyclo (Arg-Gly-Asp-d-Phe-Lys); AE105 = Ac-Lys-Gly-Asp-Cha-Phe-(D)Ser-(D)Arg-Tyr-Leu-Trp-Ser-NH_2_; JMV594 = (D)Phe-Gln-Trp-Ala-Val-Gly-His-Sta-Leu-NH_2_.

^*c*^2-AET is 2-aminoethanethiol; NOTA is 2-S-(4-isothiocyanatobenzyl)-1,4,7-triazacyclononane-1,4,7-triacetic acid; Cy5.5 is cyanine 5.5.

In comparison with previous synthetic strategies, this approach demonstrated two major advantages: (1) the PA scaffold is very simple and available for controllable mono-thiol-addition or double-thiol-addition with high regio-selectivity (Table 2); (2) the reaction can tolerate a wide range of functional groups such as –NH_2_, –CO_2_H and –OH, and the protecting-group-free strategy can be easily realized for the integration of dual-modality probes in a simple and straightforward way.

**Fig. 2:**
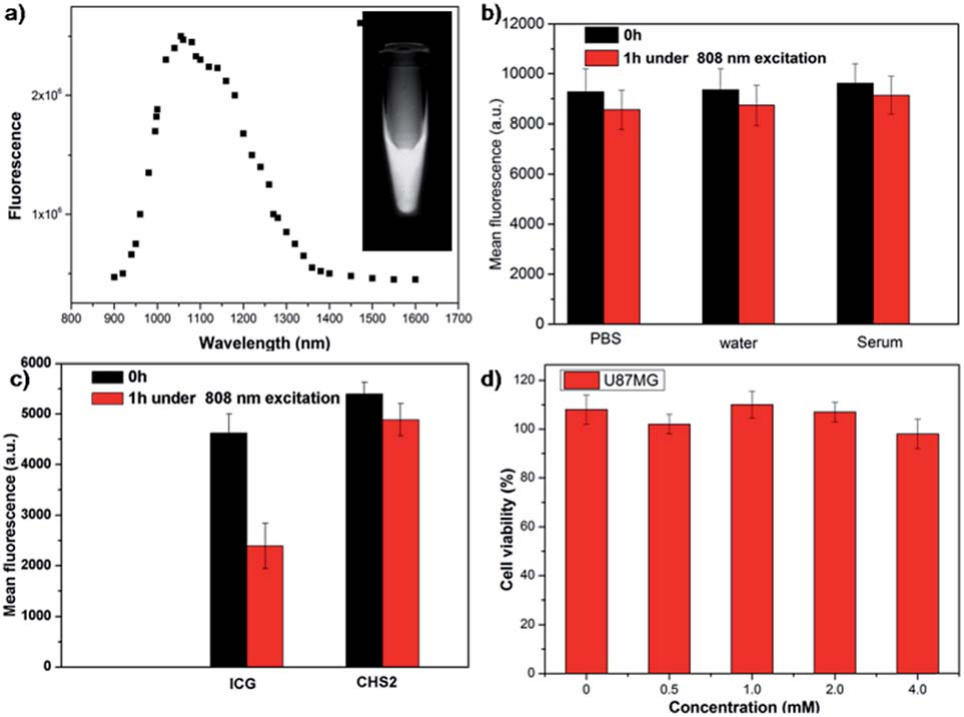
(a) Fluorescence emission of **CHS2** under 808 nm excitation; (b) photostability of **CHS2** in water, PBS and serum under 808 nm laser illumination (105 mW cm^–2^) for 1 h; (c) photostability of ICG *vs.***CHS2** under 808 nm laser illumination (82 mW cm^–2^) for 1 h; (d) cellular toxicity of different concentrations of **CHS2** in U87MG cell lines.

The first peptide-based NIR-II/PET dual-modal imaging probe **CHS2** was successfully generated *via* the above methodology. **CHS2** was purified by HPLC and characterized by MALDI-TOF MS analysis [calcd for: C_159_H_200_N_35_O_37_S_5_^+^ ([M + H]^+^): 3351.3, found: *m*/*z* 3350.2 (Fig. S10†)]. The UV-vis-NIR absorption band of the **CHS2** probe was at 600–900 nm (Fig. S11†). Meanwhile, the fluorescence emission spectrum of the **CHS2** probe indicated a maximum wavelength at 1055 nm in PBS buffer (Fig. 2a and S12†). The fluorescence quantum yield of the **CHS2** probe was ∼0.20% in water, measured against an IR-26 reference with a nominal quantum yield of 0.5% under 785 nm excitation. **CHS2** also exhibited high photostability in PBS, water and mouse serum with negligible decay under continuous excitation for 1 h (Fig. 2b). However, NIR-I probes such as ICG decayed ∼50% under the same conditions (Fig. 2c). Furthermore, the high viability of U87MG cell lines after 24 h incubation with different concentrations of **CHS2** and ^nat^Ga-**CHS2** demonstrated the high biocompatibility of **CHS2***in vitro* (Fig. 2d and S13†). Briefly, **CHS2** was then incubated with ^68^Ga [2 mCi] under mild conditions for 15 min according to a previously reported method,^41^ and purified by RP-HPLC resulting in over 95% purity (Fig. S14†). The specific activity of **^68^Ga-CHS2** was determined to be ∼25 GBq μmol^–1^ and it demonstrated excellent stability in mouse serum (no release of ^68^Ga for 2 h). U87MG cells were chosen to investigate the specificity of **^68^Ga-CHS2** toward integrin αvβ_3_.^42^,^43^ As shown in Fig. S15,† **^68^Ga-CHS2** exhibited good uptake in U87MG cells. In the blocking group, the cells were incubated with **^68^Ga-CHS2** (1 μCi per well) and unlabeled RGD (2 μg per well) as a blocking agent, and the uptakes were significantly reduced, indicating that **^68^Ga-CHS2** can specifically bind to integrin αvβ_3_ receptors on U87MG cells. Thus, the properties of **^68^Ga-CHS2** warrant further *in vivo* applications.

**Fig. 3:**
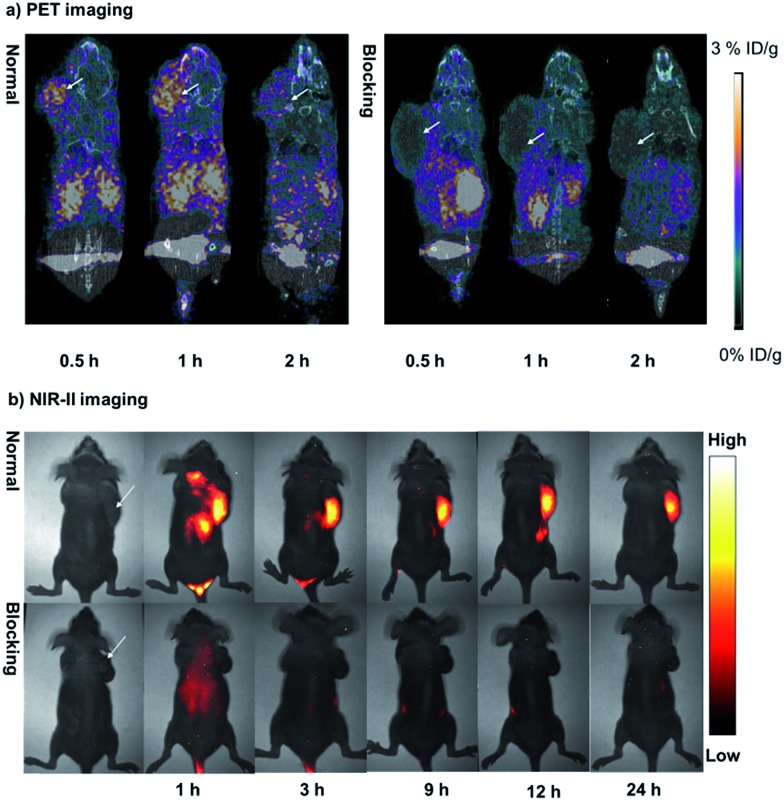
(a) The PET/CT images of U87MG-tumor-bearing mice (white arrows indicate the location of the tumor, *n* = 3 per group) acquired at 0.5, 1, and 2 h after tail vein injection of **^68^Ga-CHS2** with and without the blocking agent RGD. (b) NIR-II images of the U87MG tumor at 1, 3, 9, 12, and 24 h after tail vein injection of **CHS2** with and without the blocking agent RGD under 808 nm excitation (82 mW cm^–2^), 1000 LP and 40 ms.

The nude mice bearing U87MG xenografts (*n* = 3) were injected with ∼85 μCi (1 nmol) of **^68^Ga-CHS2** and were imaged with micro-PET/CT and NIR-II separately. As shown in Fig. 3a and b, at different time points during the scan, the tumor could be clearly visualized on both PET and NIR-II images with a high signal to background ratio (T/N). The *in vivo* specificity of the dual-modal imaging probe was confirmed by the blocking experiment and the intensity of the tumor signals was significantly reduced after co-injection of unlabeled RGD (750 μg per mouse) for both PET and NIR-II imaging. The quantitative analysis of the PET images further revealed that the tumor uptake values of **^68^Ga-CHS2** were gradually increased from 0.5 to 1 h, and then reduced at 2 h after injection, with 0.77 ± 0.13, 2.48 ± 0.32 and 2.14 ± 0.27% ID g^–1^ at 0.5, 1 and 2 h, respectively (Fig. S16†). In contrast, significantly lower tumor uptake values were observed for the blocking group with values of 0.32 ± 0.07, 0.28 ± 0.05 and 0.18 ± 0.04% ID g^–1^ at 0.5, 1 and 2 h, respectively. From the NIR-II imaging data, the U87MG tumor fluorescence signals were successfully reduced at later time points in the blocking group. The T/N ratios obtained by NIR-II imaging for **CHS2** are shown in Fig. S17,† and are proven to be much higher than those of the blocking group at all time points. Moreover, the highest tumor contrast was obtained at the 12 h time point with a T/N value of 4.77 ± 0.26, which is 2-fold more than previously reported NIR-I/RGD based probes.^27^ Biodistribution studies were evaluated for this dual-modal probe in major organs (Fig. S18 and S19†). Pharmacokinetics of **^68^Ga-CHS2** demonstrated renal and hepatobiliary excretion, with a trace amount of **CHS2** remaining in the kidney and liver for 60 h post-injection (PI) (Fig. S19 and S20†). These results also matched well with PET and NIR-II imaging data. Thus, a powerful synergy can be achieved by combining radiotracers for the detection of tumor tissue, and NIR-II optical tracers for subsequent accurate delineation of tumor lesions and resection margins.

**Fig. 4:**
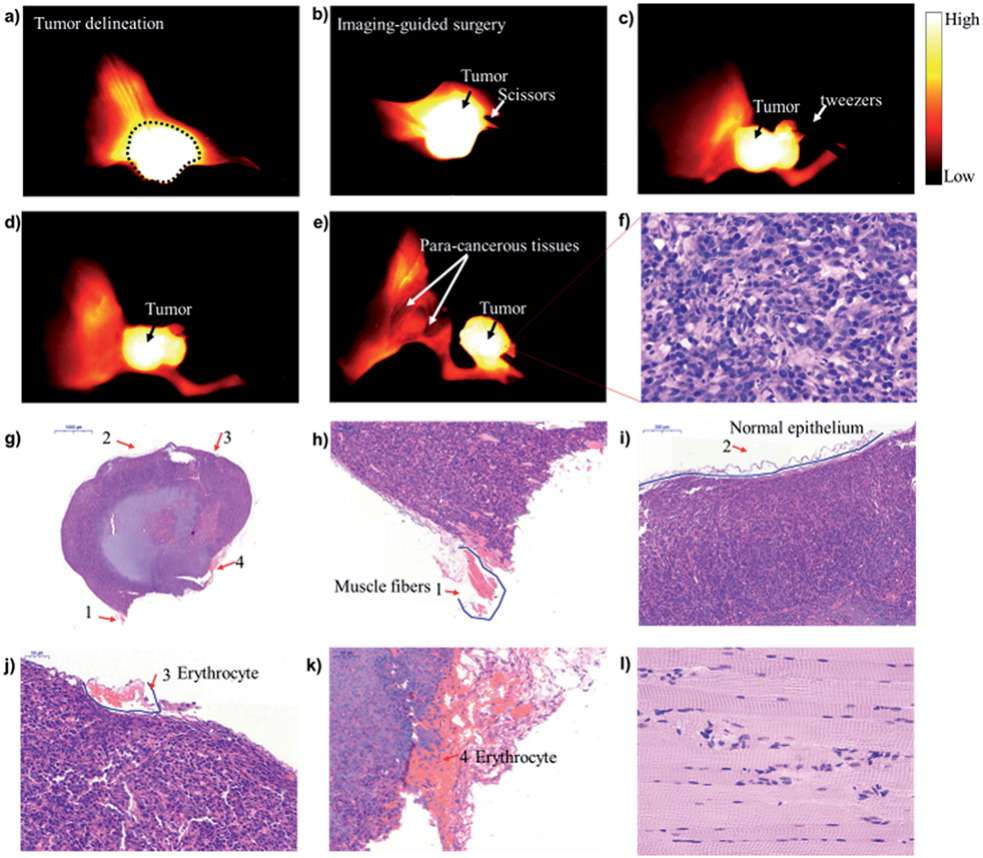
(a–e) U87MG tumor delineation and image-guided surgery at 12 h after the tail vein injection of **CHS2** under 808 nm excitation (82 mW cm^–2^), 1000 LP and 23 ms. (f) The tumor cells in this resected specimen were confirmed by high-power magnification (400×) of histological sections; (g) H&E-staining of the whole tumor. (h) The little muscle fibers marked as 1 were adjacent to the tumor tissue; (i) the brown band marked as 2 is the normal epithelium of the margin of the resected specimen; (j and k) the pink erythrocyte marked as 3 and 4 indicates the vessels in the margin of the tumor; (l) histological analysis of the excised para-cancerous tissue at 400× magnification. Cancer histologic characteristics were not detected in this para-cancerous tissue.

Encouraged by these promising results obtained *in vivo* PET/NIR-II images, **CHS2** was further evaluated for subsequent precise and accurate delineation of tumor lesions, resection margins and image-guided surgery. U87MG tumor-bearing mice (*n* = 3) were injected with 100 μg of **CHS2** and the tumor delineation could be notably identified from the surrounding background tissue at 4 h (Fig. S21†). The T/N ratio reached 4.75 ± 0.22 at 12 h post-injection, and when the tumor was dissected and removed from the soft tissue in the leg region, the T/N ratio dropped to 1.16 ± 0.27, indicating that the tumor was thoroughly dissected (Fig. 4a–e). As NIR-II fluorescence image-guided surgery was carried out, all fluorescent surgical specimens including the whole tumor and para-cancerous tissues (marked as 1–4, right adjacent to the tumor tissue) were stained with hematoxylin and eosin assessed (Fig. 4f–l). Histological analysis of excised para-cancerous tissues at different magnifications has shown that the histological characteristics of cancer were not detected. The data further verified tumor delineation and resection margins, as well as fluorescence signals by NIR-II imaging (Fig. 4f–l). This way, tumor nodules that were missed by conventional surgery but were successfully detected by our fluorescent probe **CHS2** can be analyzed to assess the sensitivity and specificity of the dual-modality intraoperative imaging approach at the histological level (Fig. 4). Our gamma probe activity measurements and NIR-II fluorescence imaging data obtained during surgery along with these hematoxylin–eosin staining results could lead to a reduced time of examination, which may aid the surgeon in making better decisions during the surgery.

## Conclusions

In summary, we have successfully developed a base-catalyzed strategy for the concise construction of an integrin αvβ_3_-targeted dual-modality imaging probe, **^68^Ga-CHS2**, for tumor delineation and image-guided surgery. This probe has demonstrated excellent imaging characteristics *in vivo* and has high clinical and translational potential. Our highly innovative approach to obtain tumor-free resection margins or maximal cytoreduction using specific tumor targeting dual-label tracers that can be detected with a **^68^Ga** probe and a fluorescence NIR-II camera during surgery will affect patient survival. Thus, our first targeted dual-modality NIR-II/PET image-guided surgery results show the potential to take oncological surgery one step further and may ultimately contribute to the improved survival of cancer patients.

All animal studies were performed in accordance with the Guidelines for the Care and Use of Laboratory Animals of the Chinese Animal Welfare Committee and approved by the Institutional Animal Care and Use Committee (IACUC), Wuhan University Center for Animal Experiment, Wuhan, China.

## Author contributions

X. C. Hong and Z. Cheng conceived and designed the experiments. Y. Sun, X. D. Zeng, Y. L. Xiao, C. H. Liu, H. Zhu and H. Zhou performed the experiments. Z. Y. Chen, F. C. Xu, J. L. Wang, M. Y. Zhu, J. Z. Wu, M. Tian, H. Zhang, Z. X. Deng, Z. Cheng and X. C. Hong analyzed the data and wrote the manuscript. All authors discussed the results and commented on the manuscript.

## Conflicts of interest

There are no conflicts to declare.

## Supplementary Material

Supplementary information
