# Supplementary material for: Novel dual-function near-infrared II fluorescence and PET probe for tumor delineation and image-guided surgery
Source: Chem Sci. 2018 Jan 8;9(8):2092–7. doi: 10.1039/c7sc04774f (PMC5892408; doi:10.1039/c7sc04774f)
Supplement: Supplementary file 1 [file SC-009-C7SC04774F-s001.pdf]

Electronic Supplementary Material (ESI) for Chemical Science.
This journal is © The Royal Society of Chemistry 2018

Supporting Information

Novel Dual-Function Near-Infrared II Fluorescence and PET Probe for Tumor Delineation and Image-Guided Surgery

Yao Sun, Xiaodong Zeng, Yuling Xiao, Changhao Liu, Hua Zhu, Hui Zhou, Ziyang Chen, Fuchun Xu, Jule Wang, Mengyue Zhu, Junzhu Wu, Hong Zhang, Zixin Deng, Zhen Cheng*and Xuechuan Hong*

Table of Contents

Figure S1 - Figure S21	S2-S22
Table S1 - Table S2	S23
General methods	S24
Absorption spectrum, PL excitation spectra and fluorescence quantum yield	S25
Radiochemistry and in vitro metabolic stability	S25
Cell line and animal model, Cell uptake, Cell viability	S25-S26
In vivo PET imaging of tumors	S26
In vivo NIR-II fluorescence imaging	S27
Ex vivo biodistribution analysis	S27
Histological Analysis	S27
Chemical synthesis and characterization	S27-S37
^{1}H and ^{13}C NMR for **1**	S38
^{1}H and ^{13}C NMR for **1c**	S39
^{1}H and ^{13}C NMR for **1d**	S40
^{1}H and ^{13}C NMR for **4**	S41
^{1}H and ^{13}C NMR for **5**	S42
References	S43

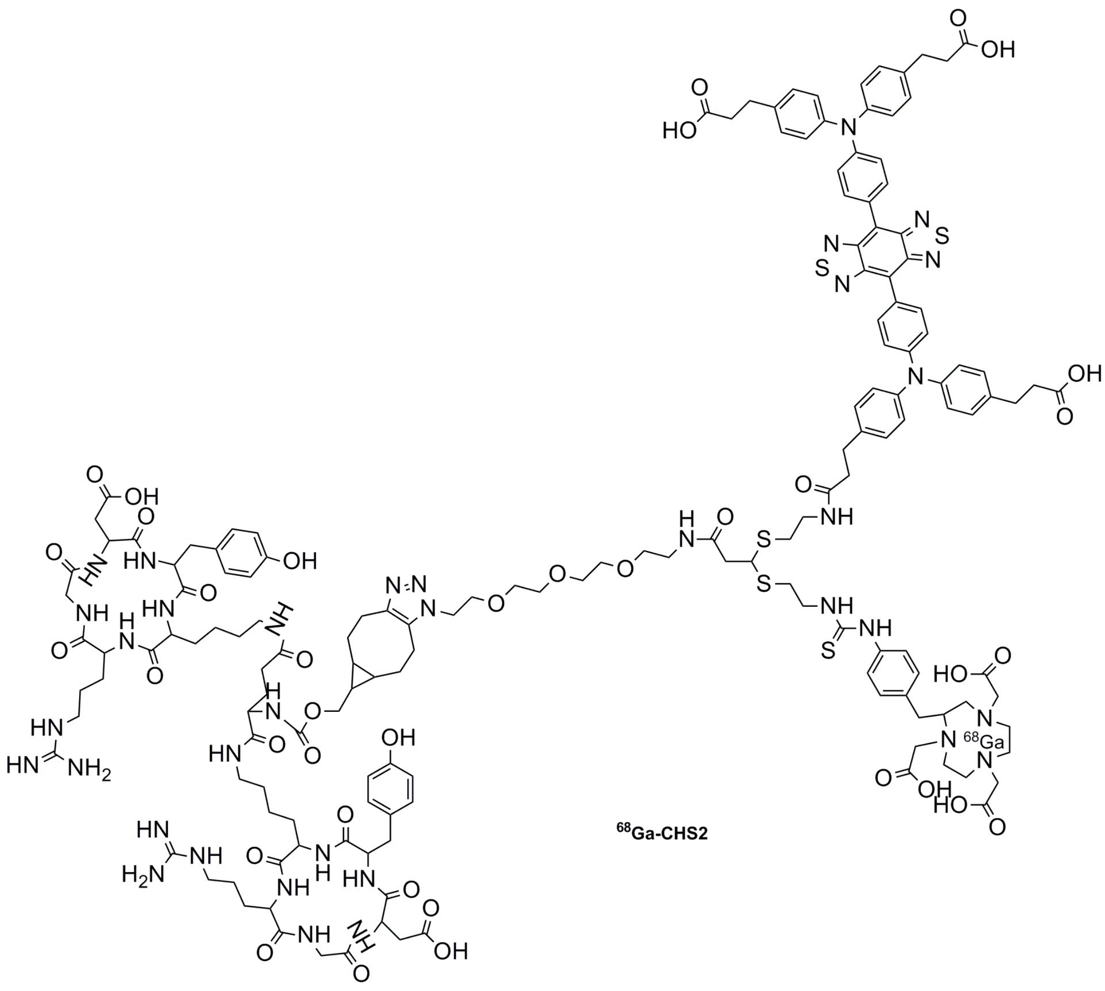

Figure S1. The chemical structure of ^{68}Ga-**CHS2**

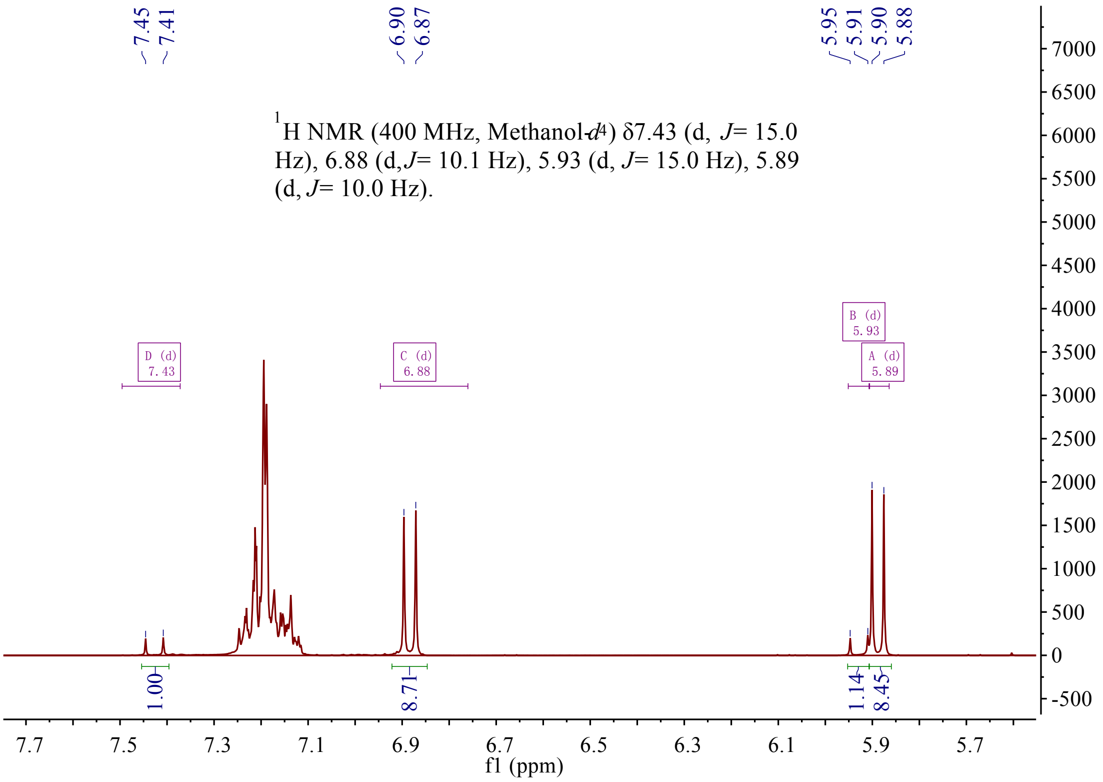

Figure S2. ^{1}H-NMR spectrum of **1a**. The data show that mono-adduct is the desire product and cis isomer is the main product (cis/trans= 90:10).

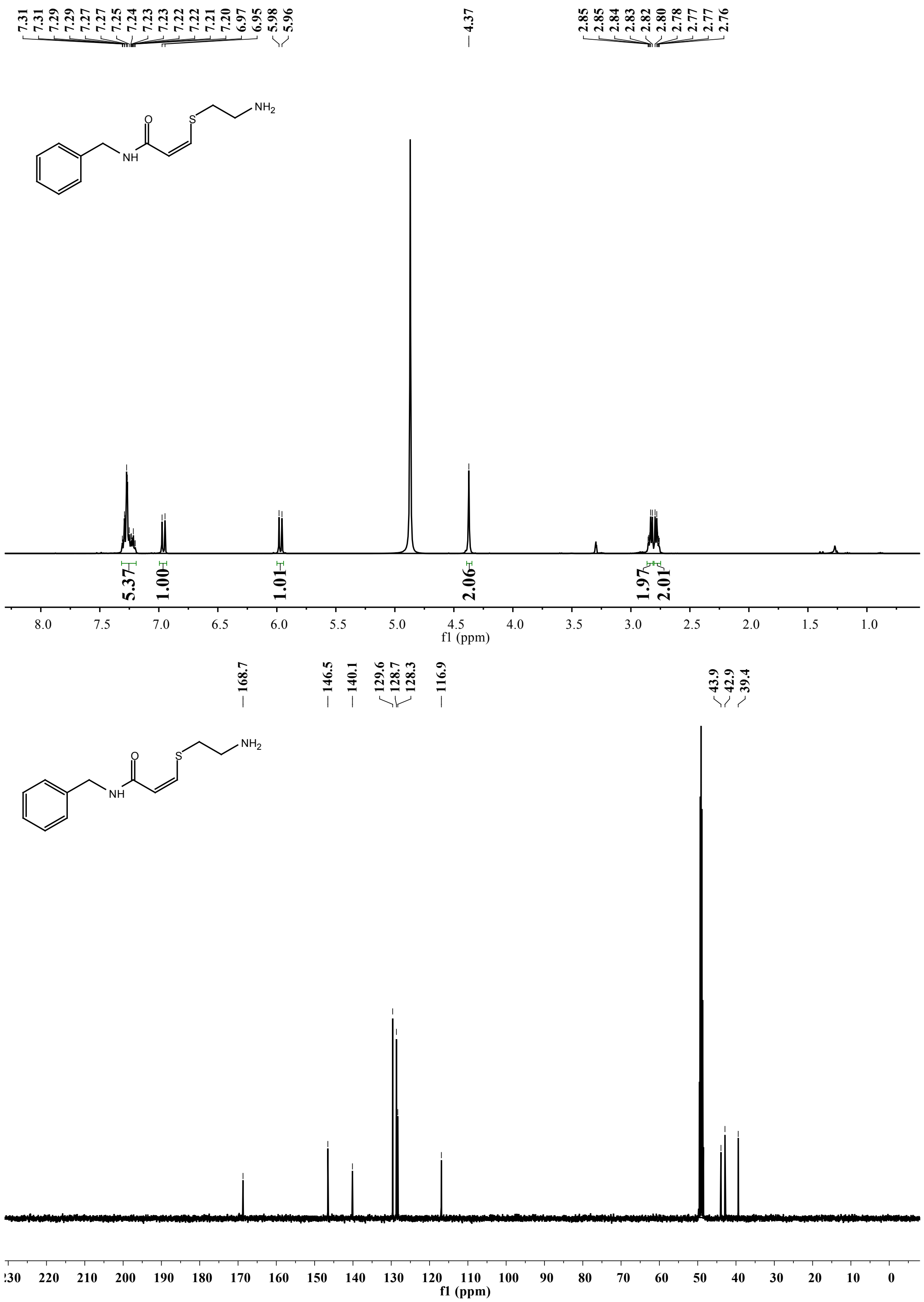

Figure S3. $^{1}H/^{13}C$NMR spectrum of cis isomer of **1a**.

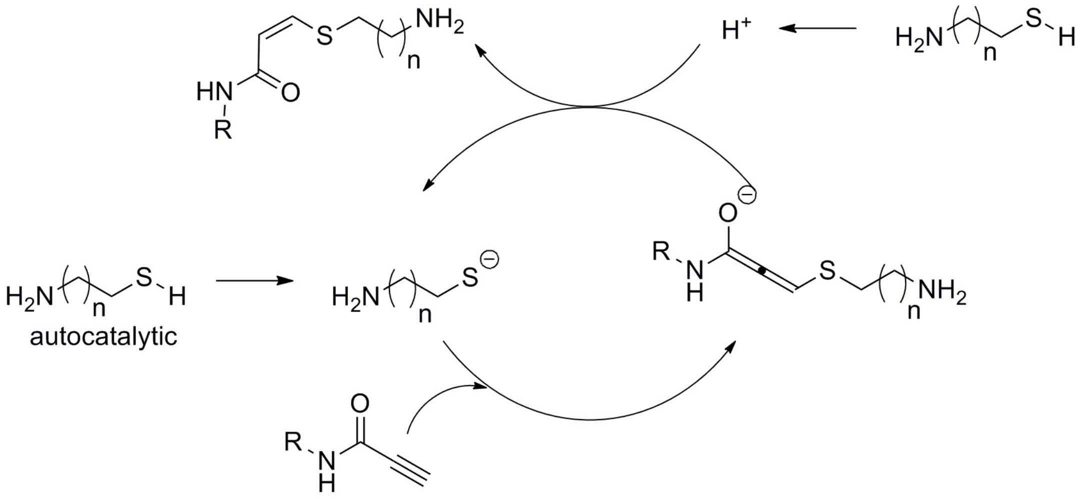

Figure S4. A possible base-catalyzed thiol-addition mechanism.

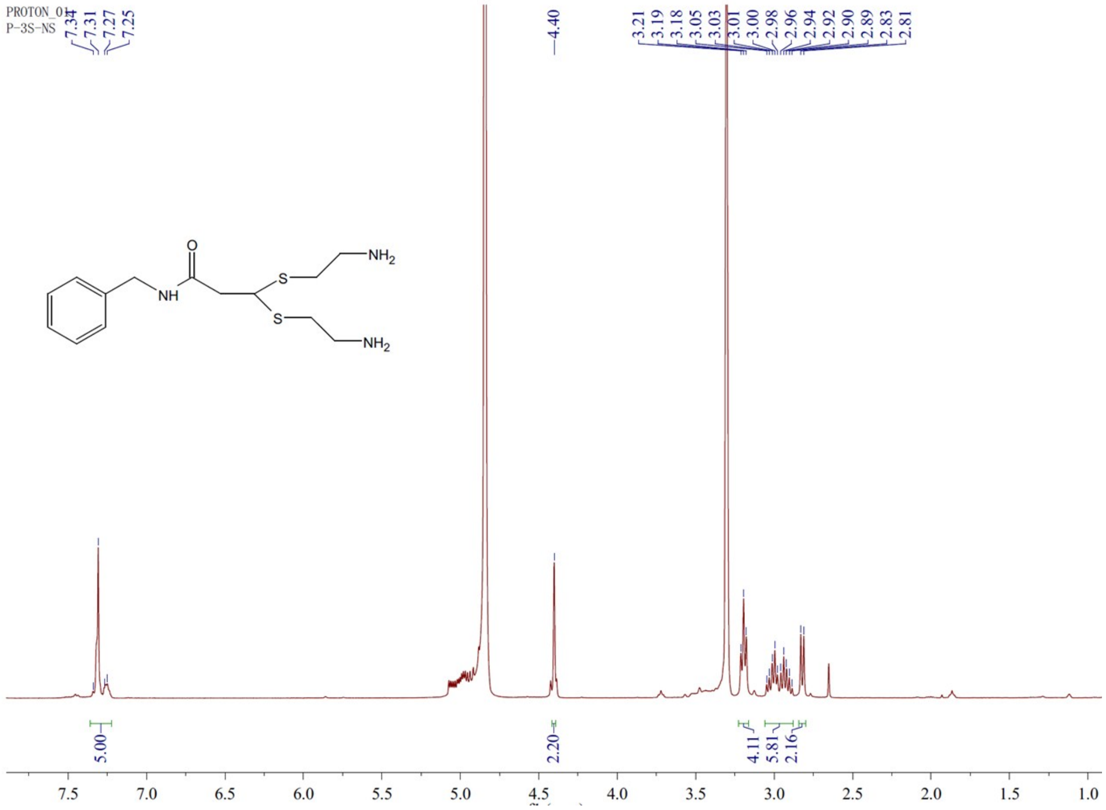

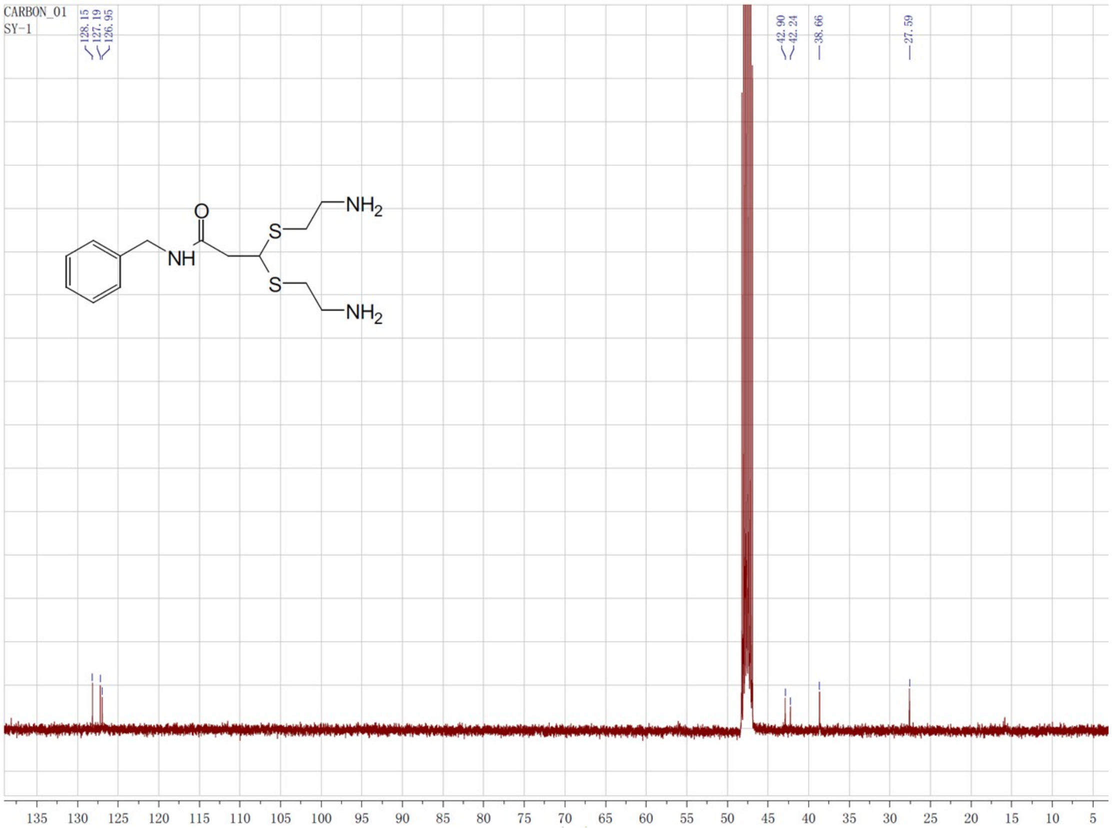

Figure S5. $^{1}H/^{13}C$NMR spectrum of **1b**.

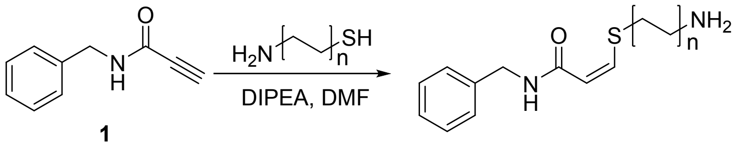

1

1c-1d
Major product

n = 3	60 min, 74%
n = 5	60 min, 68%

Figure S6. The investigation of the length of carbon linker influence of base-catalyzed thiol-addition reaction.

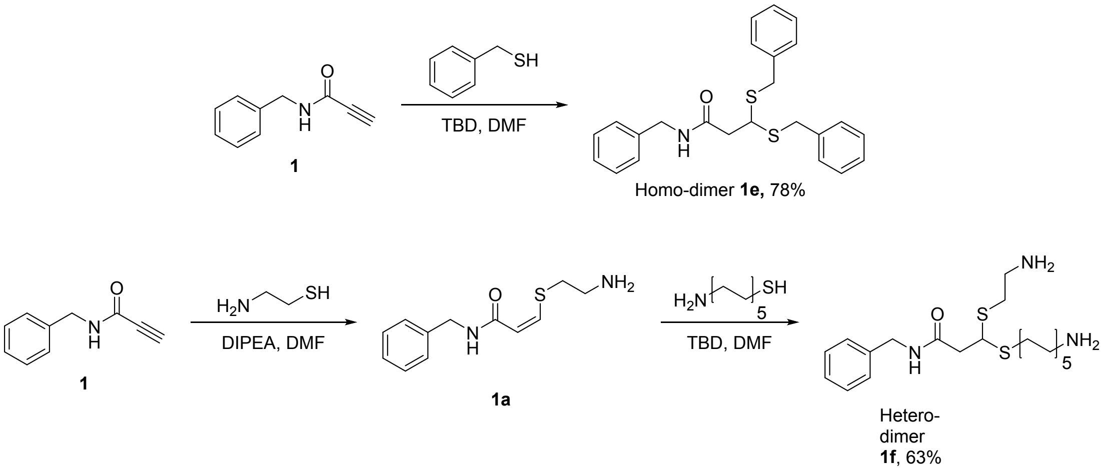

Figure S7. The construction of homo-dimer and hetero-dimer products *via* base-catalyzed thiol-addition reaction.

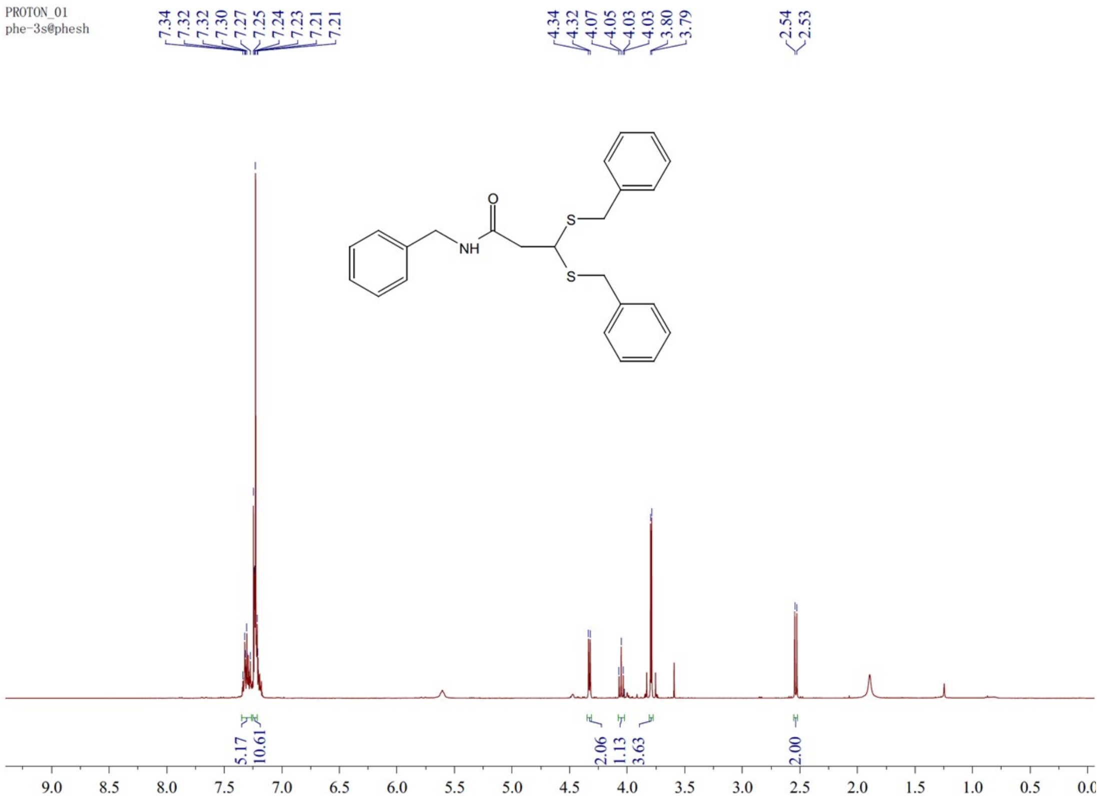

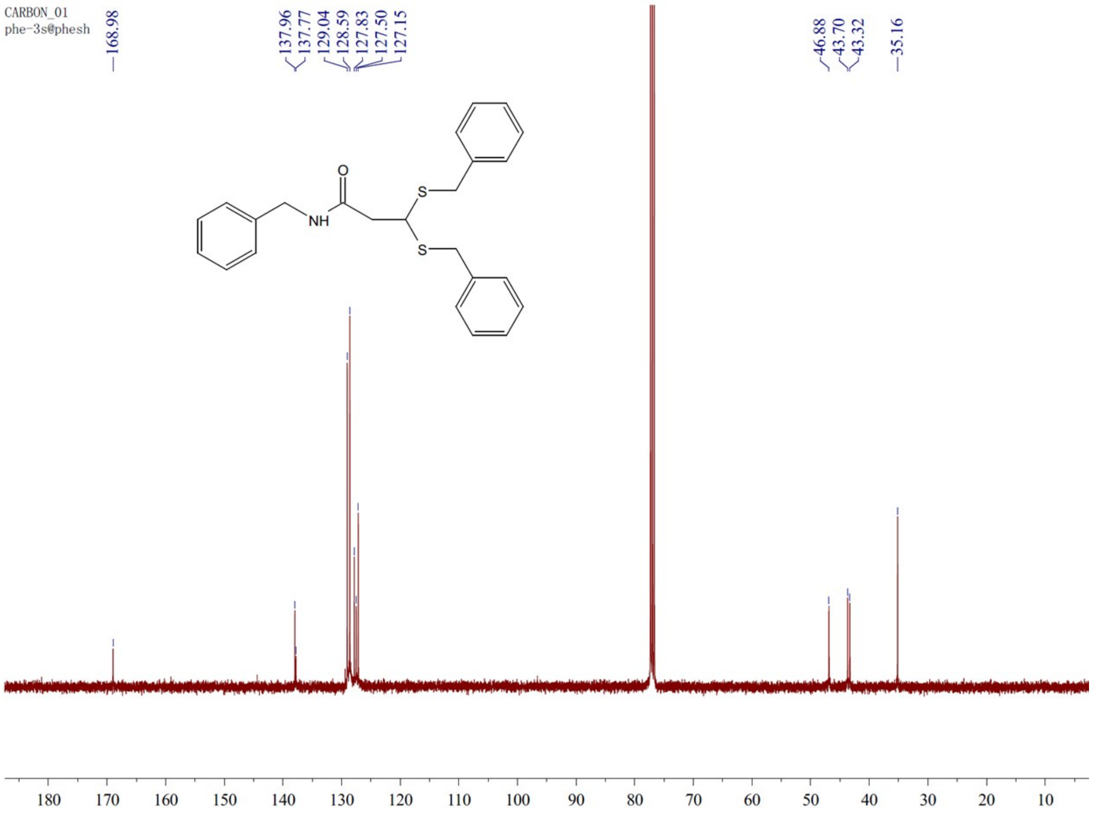

Figure S8. $^{1}H/^{13}C$NMR spectrum of **1e**.

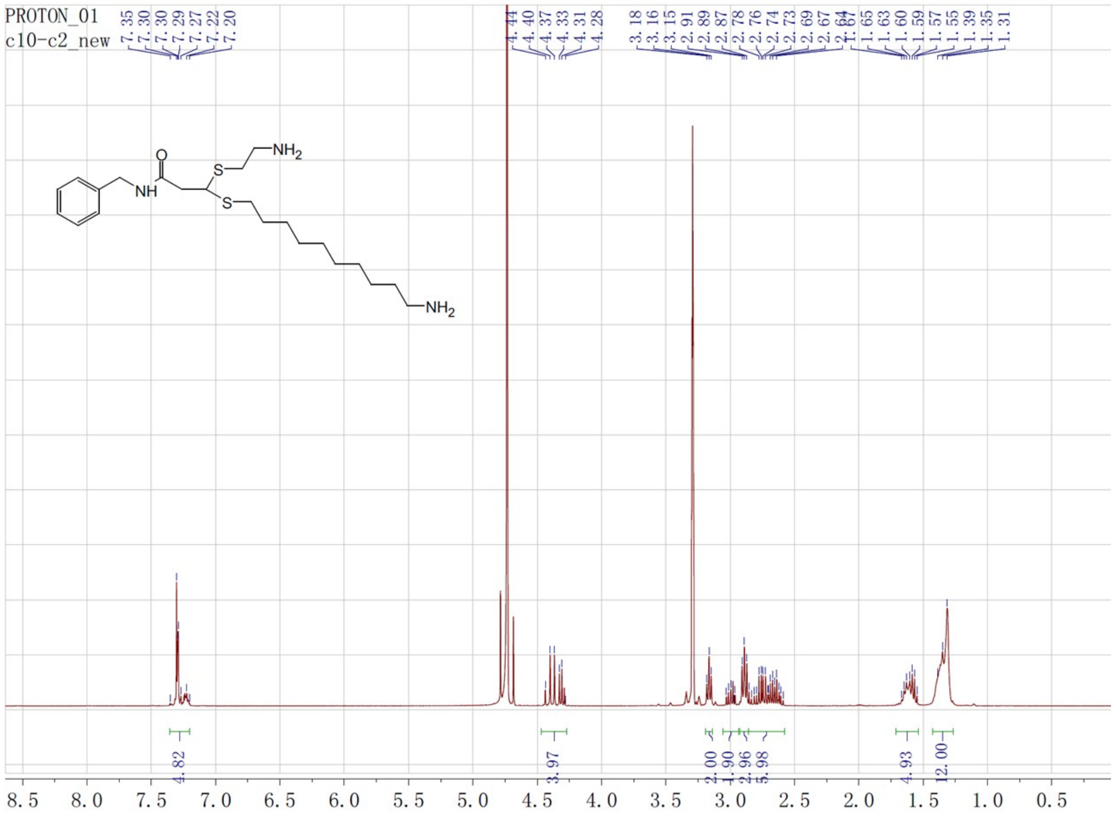

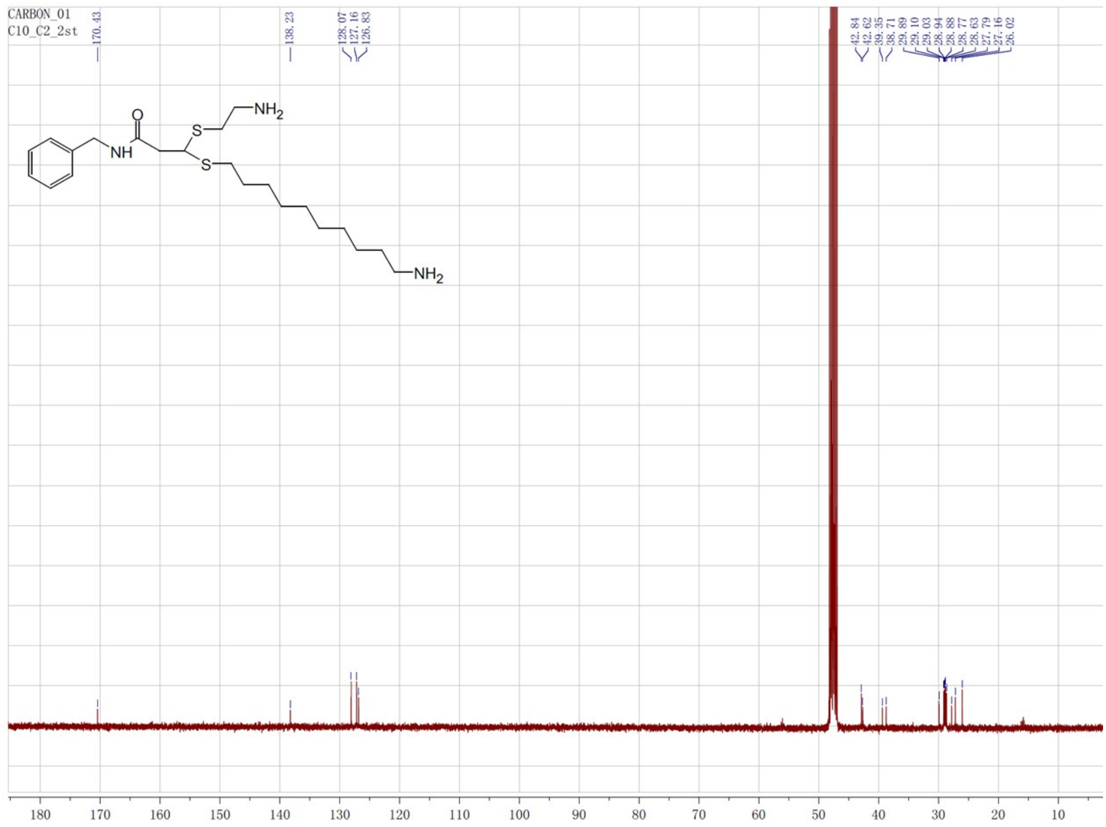

Figure S9. $^{1}H/^{13}C$NMR spectrum of **1f**

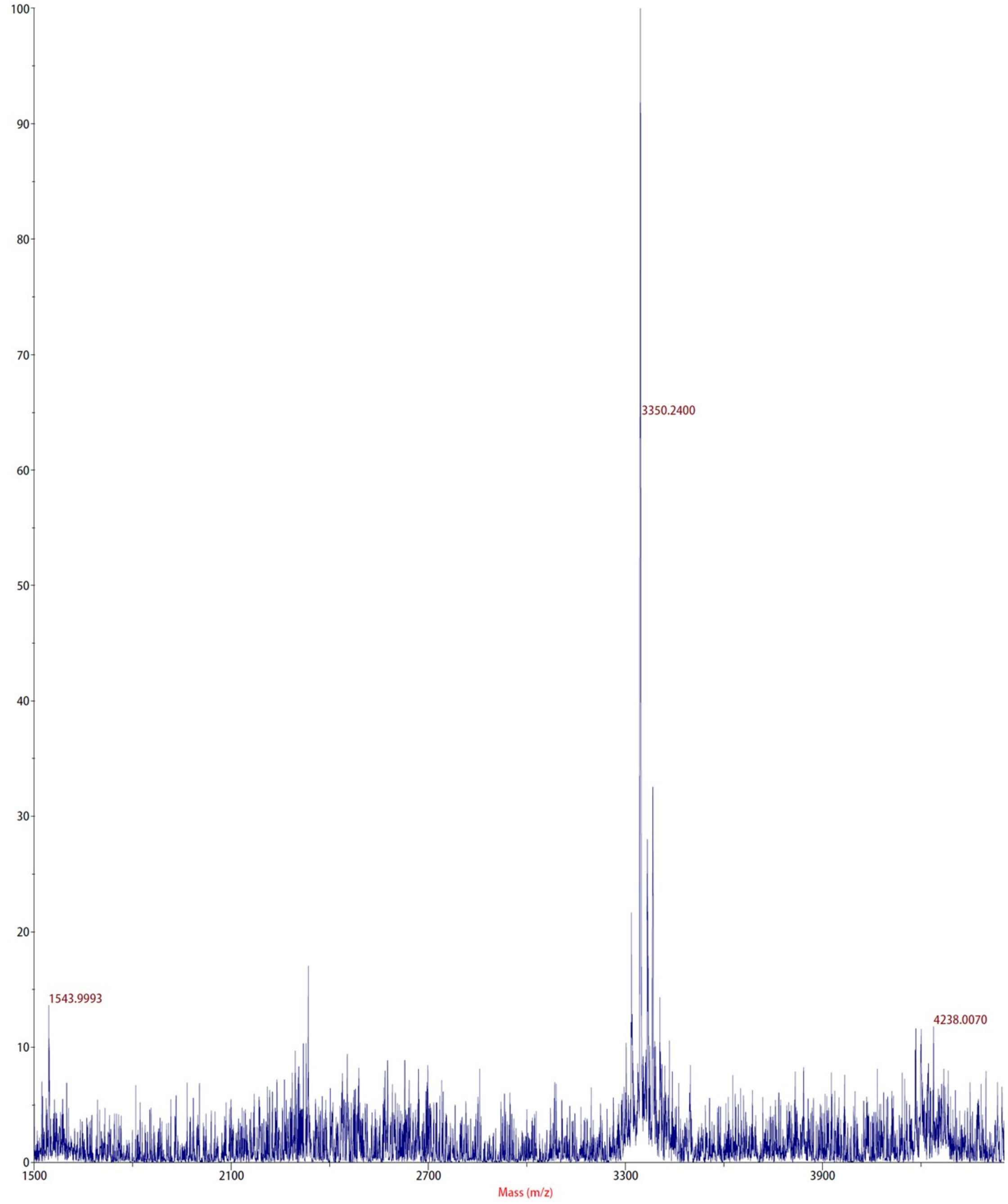

Figure S10. MALDI-TOF-MS of **CHS2**

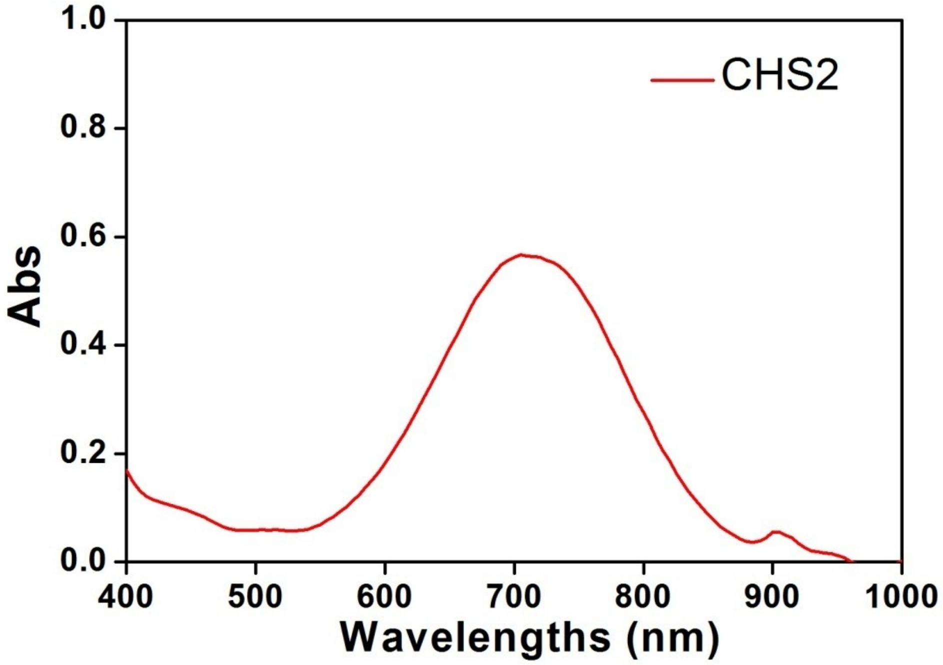

Figure S11. The absorption spectrum of **CHS2**

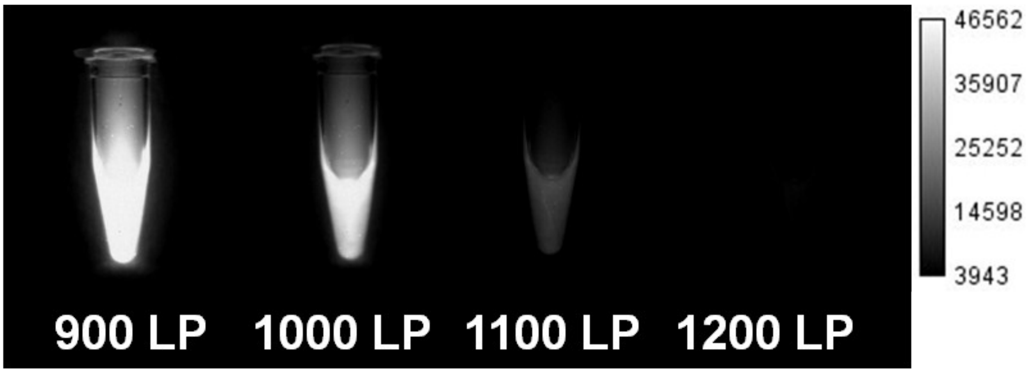

Figure S12. Fluorescent signals of **CHS2** with various long-pass (LP) filters (900-1200 nm)

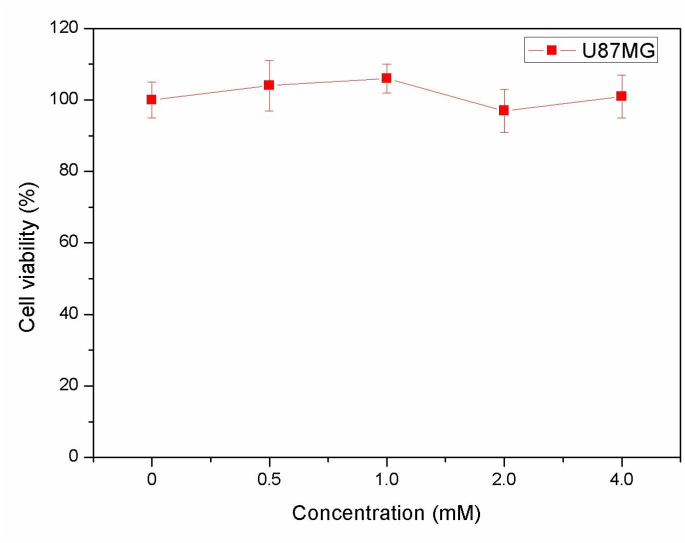

Figure S13. Cellular toxicity of different concentration of **^{nat}Ga-CHS2** in U87MG cell lines.

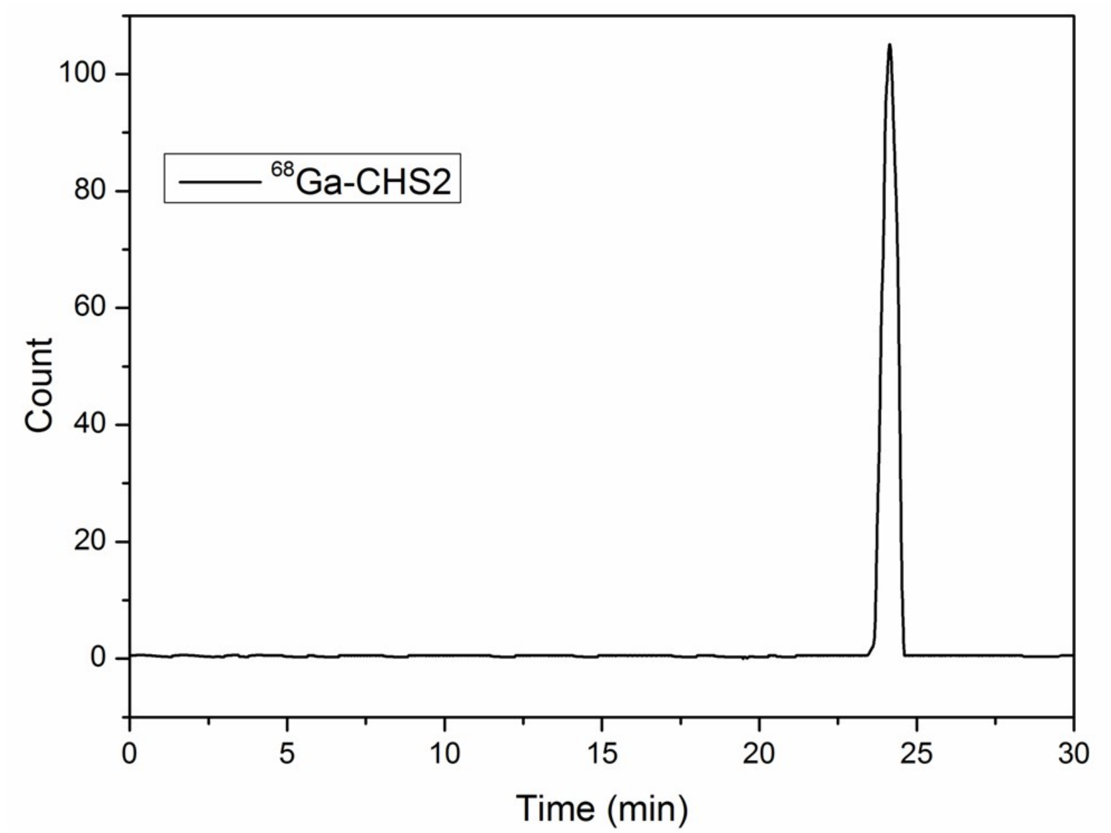

Figure S14. Radio-HPLC analysis of purified **^{68}Ga-CHS2**

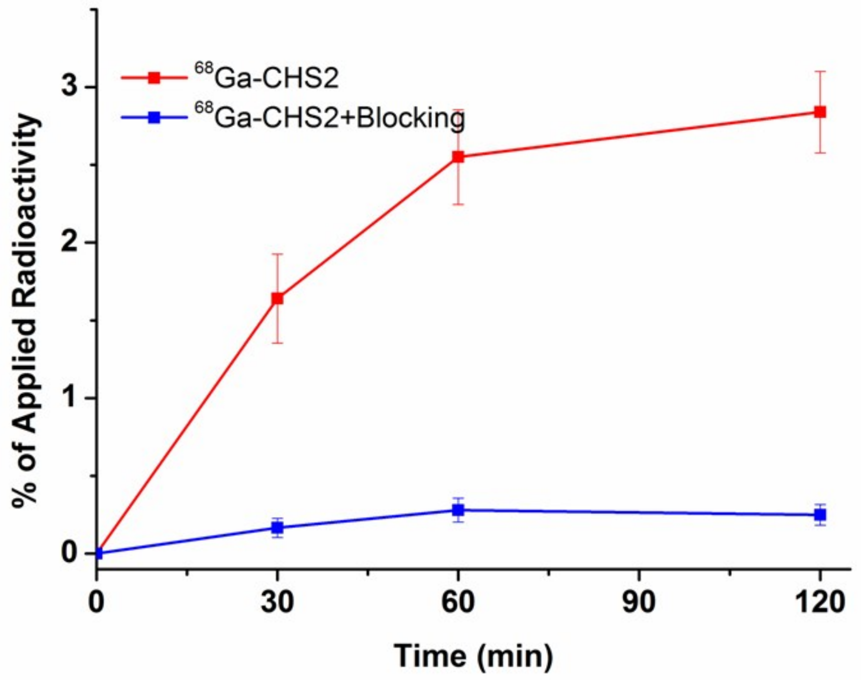

Figure S15. *In vitro* U87MG cell uptake for **^{68}Ga-CHS2** with or without RGD blocking agent

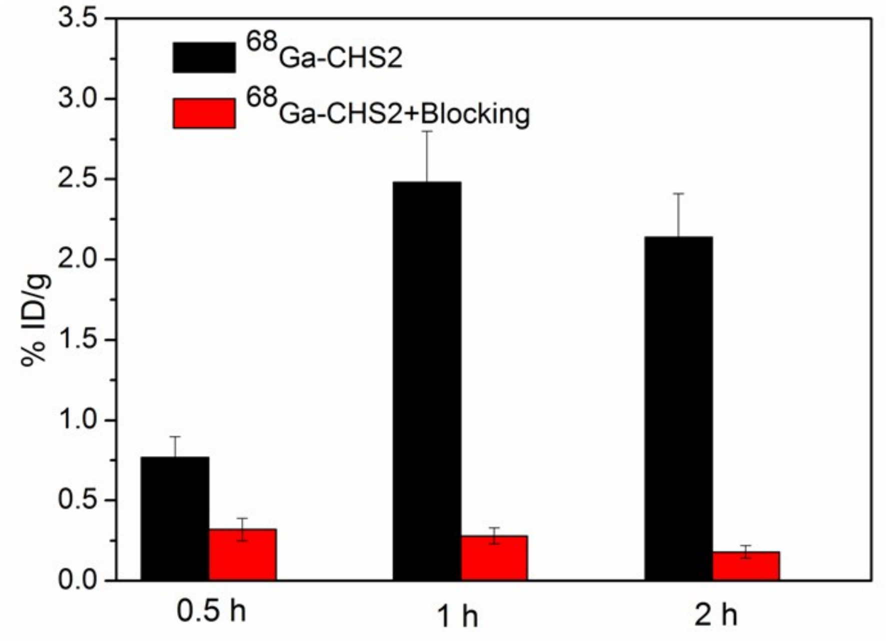

Figure S16. Quantitative ROI analysis from PET imaging, represented as the tumor PET signal intensity-time for **^{68}Ga-CHS2** (black bar) and blocking group (red bar) at 0.5, 1, and 2h.

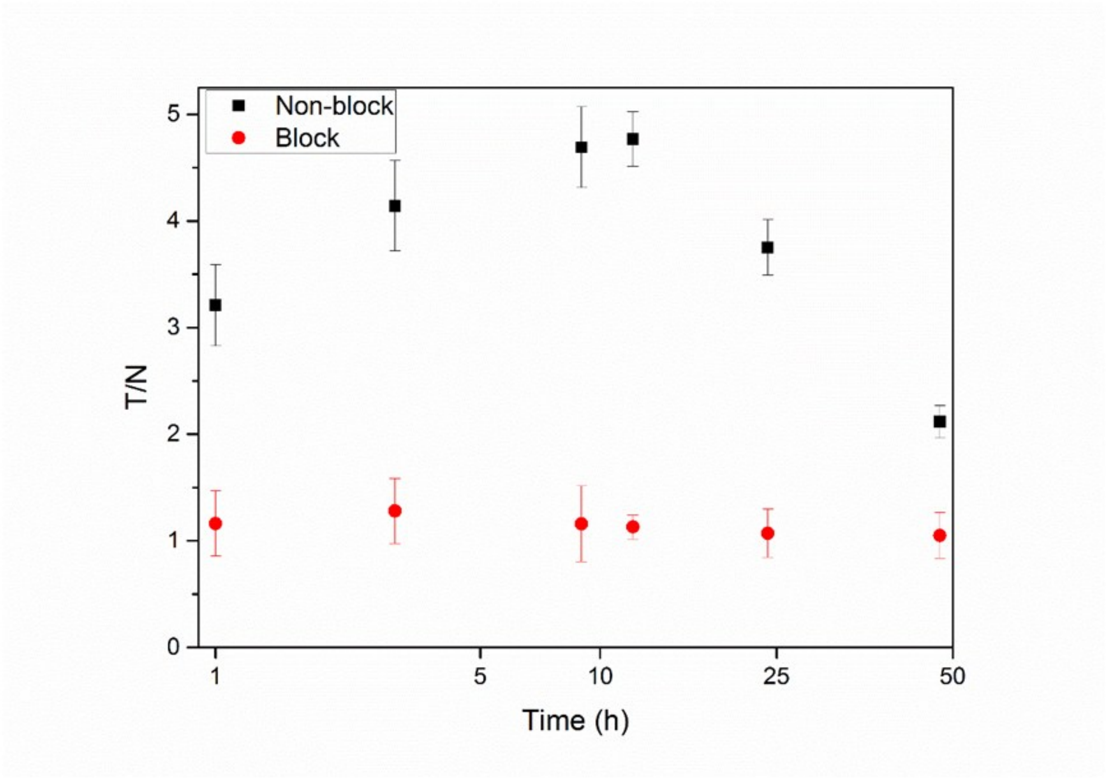

Figure S17. The NIR-II imaging, represented as (T/N) ratio for the **CHS2** probe (black bar) and blocking group (red bar) at1,3, 9, 12, 24, 48 h (under 808 nm excitation laser (82 mW/cm^2), 1000LP and 40 ms).

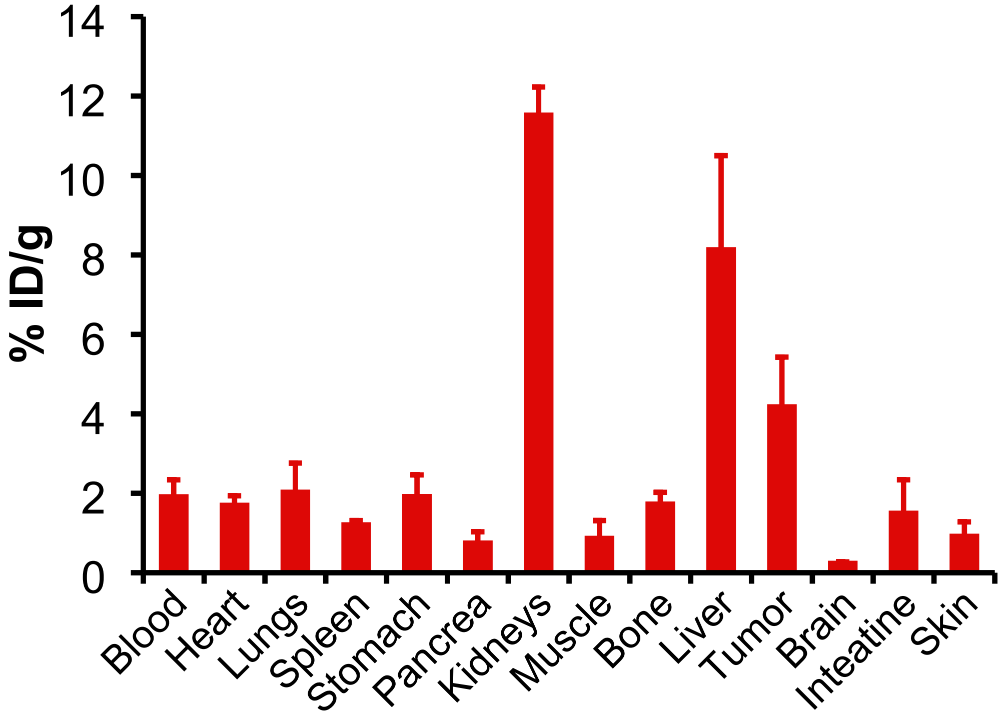

Figure S18. The biodistribution of **^{68}Ga-CHS2** in main organs at 2 h post-injection.

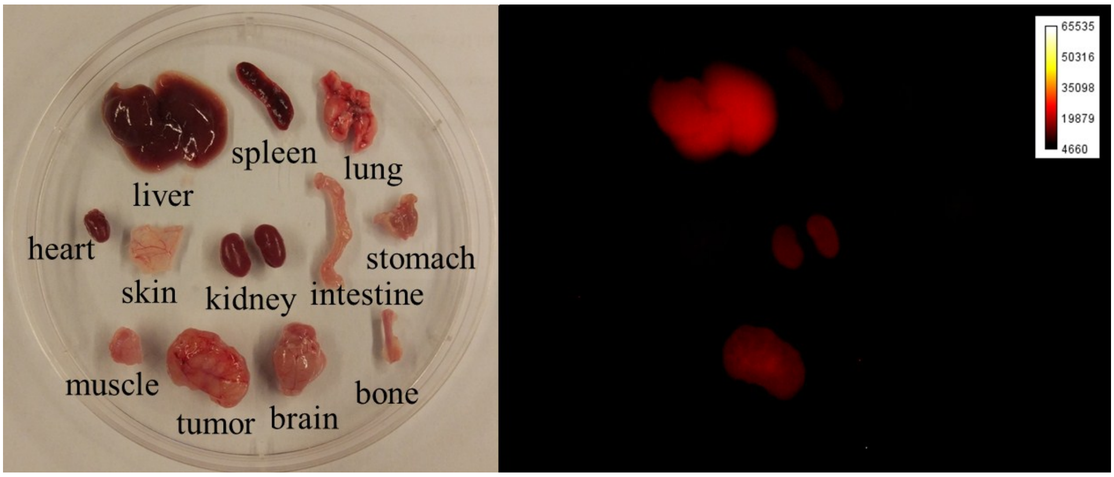

Figure S19. The biodistribution of **CHS2** in tumor mice at 60 h under 808 nm excitation laser (82 mW/cm^2), 1000LP and 20 ms.

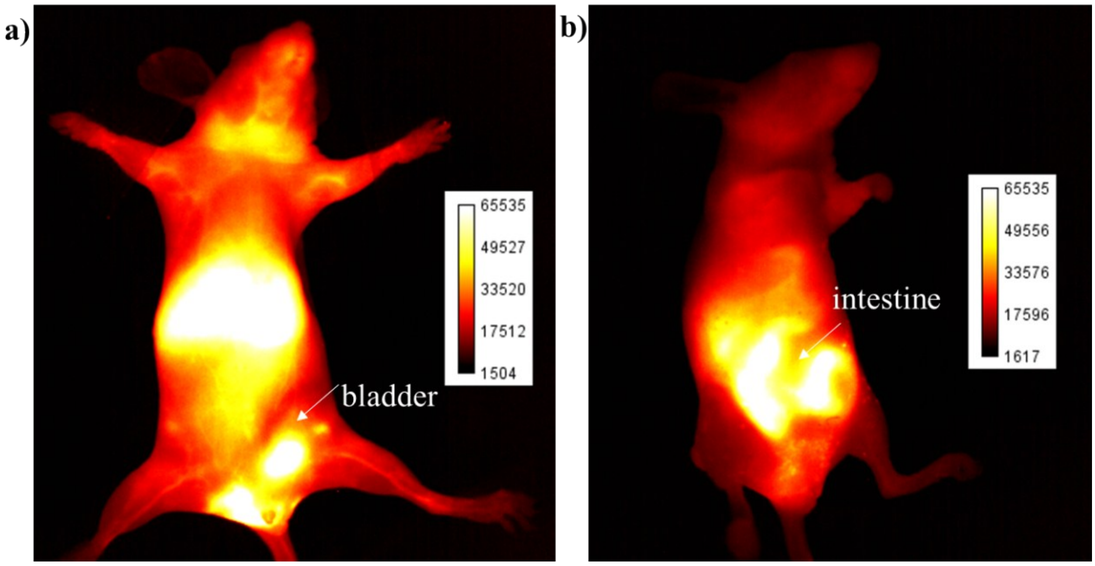

Figure S20. The NIR-II fluorescence image of tumor-bearing mice at 9 min (a) and 3 h (b) after tail vein injection of **CHS2** under 808 nm excitation laser (82 mW/cm^2), 1000LP and 15 ms (a) or 5 ms (b).

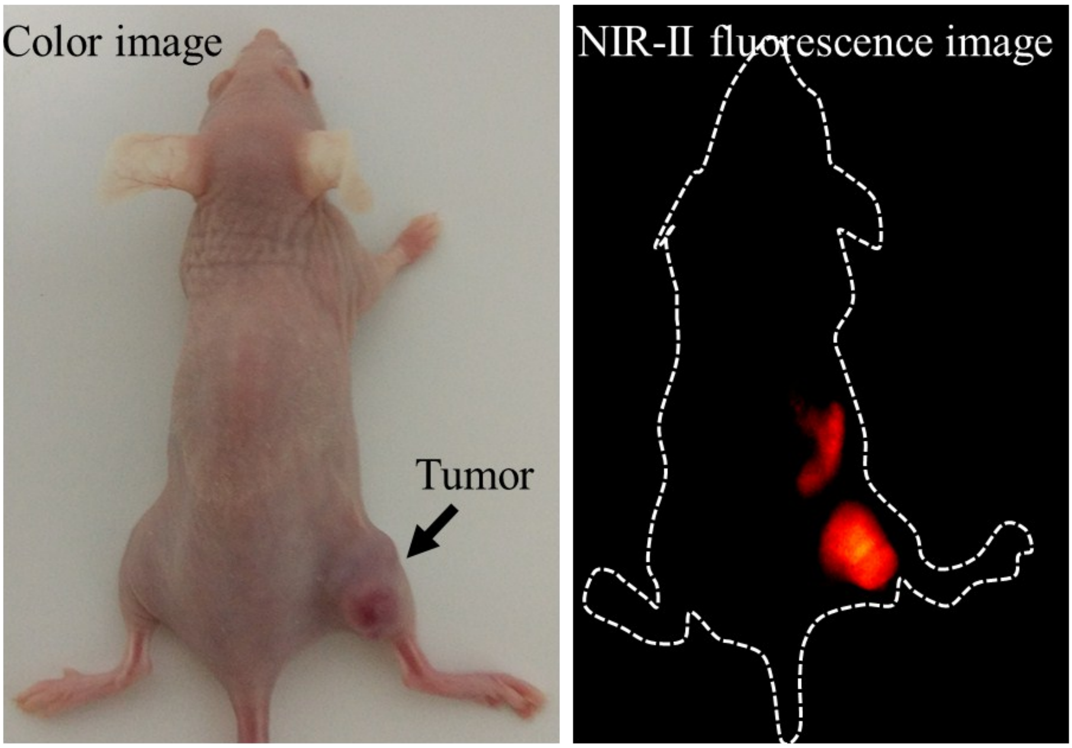

Figure S21. The color image of tumor mice and NIR-II fluorescence image of **CHS2** in tumor mice at 4 h under 808 nm excitation laser (82 mW/cm^2), 1000LP and 30 ms.

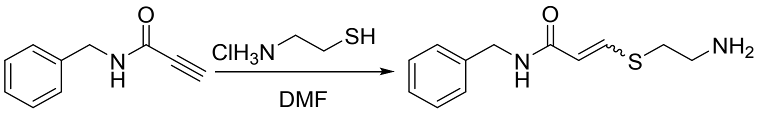

1 **1a, 5%**

Entry	Catalyst	t [min]	Thiol (equiv.)	Yield[b] (%)
1[a]	/	30	1.5 eq	5

a) the thiol is Cysteamine Hydrochloride

Table S1. Spontaneous thiol addition chemistry

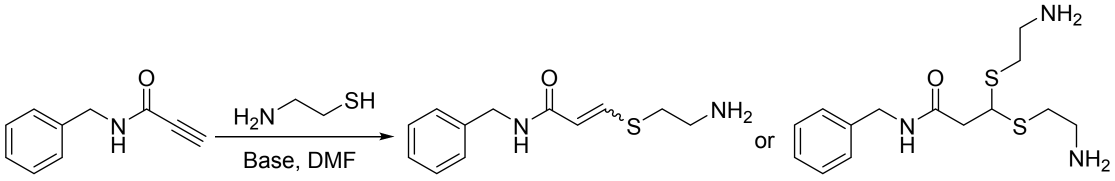

1 **1a** **1b**

Entry[a]	Catalyst	t [min]	Thiol (equiv.)	Yield[b] (%)	Product (1a/1b)
1	NEt_3 (0.1 equiv.)	30	4.0 eq	72	100:0
2	DIPEA (0.1 equiv.)	30	4.0 eq	76	100:0
3	DBU (0.1 equiv.)	30	4.0 eq	81	95:5
4	TBD (0.1 equiv.)	30	4.0 eq	70	60:40
5	TBD (0.2 equiv.)	30	4.0 eq	73	18:82
6	TBD (0.2 equiv.)	60	4.0 eq	67	0:100

a) Reaction conditions: [alkyne] = 0.25 M in solvents, catalyst = 0.1 or 0.2 equiv.
b) Isolated yield calculated on the basis of the starting alkyne and referred to the major products

Table S2. Base-catalyzed thiol double addition chemistry

General methods

All chemicals were purchased from commercial sources (such as Aldrich, conju-probe, Lumiprobe and peptide international). The ^{1}H and ^{13}C NMR spectra were acquired on a Bruker 400 MHz magnetic resonance spectrometer. Data for ^{1}H NMR spectra are reported as follows: chemical shifts are reported as δ in units of parts per million (ppm) relative to chloroform-d (δ 7.26, s); multiplicities are reported as follows: s (singlet), d (doublet), t (triplet), q (quartet), dd (doublet of doublets), m (multiplet), or br (broadened); coupling constants are reported as a *J* value in Hertz (Hz); the number of protons (n) for a given resonance is indicated nH, and based on the spectral integration values. MALDI-MS spectrometric analyses were performed on an Applied Biosystems 4700 MALDI TOF mass spectrometer. HPLC was performed on a Dionex HPLC System (Dionex Corporation) and a reversed-phase C18 column was used for analysis (Phenomenax, 5 µm, 4.6 mm × 250 mm) and semi-preparation (Agilent, 5 µm, 10 mm × 250 mm).

Absorption spectrum, PL excitation spectra and fluorescence quantum yield. UV-Vis-NIR absorbance of the probe was recorded on a PerkinElmer Lambda 25 UV-Vis spectrophotometer. PLE spectrum of the **CHS2** solution was taken using an Applied NanoFluorescence spectrometer. The fluorescence quantum yield was measured by a modified procedure according to previously published paper.[1] A series of solutions of IR26 in DCE were prepared with absorbance values at 785 nm to be ~ 0.10, ~ 0.08, ~ 0.06, ~0.04 and ~0.02. Absorption and fluorescence spectra of IR26 were measured using the instruments described previously. Same solution preparation, absorption and fluorescence spectra measurements were performed for **CHS2**. The integrated fluorescence was plotted against absorbance for both IR26 and **CHS2** and fitted into a linear function. Comparison of the slopes led to the determination of the quantum yield of **CHS2**. The quantum yield was calculated in the following manner:

$$QY_{CHS2} = QY_{IR26} \times \frac{Slope_{CHS2}}{Slope_{IR26}} \times \frac{{n_{CHS2}}^2}{{n_{IR26}}^2}$$

Where QY_{CHS2} is the QY of probe **CHS2** in water, QY_{IR26} is the QY of IR-26 in DCE, n_{CHS2} and n_{IR26} are the refractive indices of water and DCE.

Radiochemistry and in vitro metabolic stability. Fresh ^{68}Ga was eluted as $^{68}GaCl_3$ (approximately 185 MBq) obtained from the generator. Briefly, **CHS2** (1 nmol) was dissolved in

NaOAc buffer (pH=4.5) and labeled with 3.5-7.5 MBq of ^{68}Ga for 15 min at 70 ^{o}C. The ^{68}Ga labeled **CHS2** was then purified by analytic radio-HPLC. The radioactive peaks containing the desired products were collected and rotary-evaporated to remove the solvent. The products were then formulated in phosphate-buffered saline and passed through a 0.22-μm Millipore filter into a sterile vial for subsequent in vitro and in vivo experiments. The labeling yield was above 95% for all the probes. The in vitro metabolic stability of the **^{68}Ga-CHS2** was evaluated by incubation with mouse serum (0.5 mL) at 37°C. The solutions were filtered using a NanoSep 10K centrifuge (Pall Corp.) to isolated low-molecular-weight radiocomplexes. The samples were analyzed by the radio-HPLC, and the percentages of intact PET probe were determined by quantifying the peaks areas corresponding to the intact PET-probe and the degradation products.

^{nat}Ga-CHS2. A solution of $GaCl_3$ (0.1 M) in 10 μL 0.1 N HCl was added to the compound **CHS2** (0.8 μmol) in 100 μL H_2O. The mixture was stirred at RT for 1 h. The crude product was purified by HPLC to obtain ^{nat}Ga-**CHS2**.

Cell line and animal model. The cells were cultured in Dulbecco's modified Eagle's medium (DMEM) supplemented with 10% (v/v) fetal bovine serum and 1% (v/v) penicillin at 37°C and 5% CO_2. For PET imaging studies, U87MG glioblastoma cells were obtained from the American Type Culture Collection (Manassas, VA, USA) and culture media was obtained from Invitrogen Co. (Carlsbad, CA, USA). The U87MG tumor models were established by subcutaneous injection of U87MG cells (~5 × 10^6 in 100 μL of PBS) into the front flank of female or male athymic nude mice (Harlan). The mice were subjected to imaging studies when the tumor volume reached 200-500 mm^3 (about 4 weeks after inoculation). The PET animal experiments followed the Guide for the Care and Use of Laboratory Animals and were performed according to a protocol approved by the Stanford University Institutional Animal Care and Use Committee (IACUC). For NIR-II imaging studies, U87MG glioblastoma cells were a gift from Dr. Qiangbin Wang (Suzhou Institute of Nano-Tech and Nano-Bionics, Chinese Academy of Sciences) and DMEM medium was purchased from Gibco. the U87MG tumor models were established by subcutaneous injection of U87MG cells (~1 × 10^7 in 100 μL of DMEM medium) into the right hand sides or right leg of female athymic nude mice (Suzhou Belda Bio-Pharmaceutical Co.). The mice were subjected to imaging studies when the tumor volume reached 300-600 mm^3 (about 2 weeks after inoculation).

The animal experiment in NIR-II imaging studies were done in the Center for Animal Experiment of Wuhan University (Wuhan, Hubei, P.R. China), which has been accredited by Association for Assessment and Accreditation of Laboratory Animal Care International (AAALAC International).

Cell uptake. U87MG cells (~1× 10^5) were suspended in 500 μL of DMEM seeded in 12-well tissue culture plates and incubate at 37 °C for overnight. After washing with PBS, the cells were incubated with **^{68}Ga-CHS2** (1 μCi/well), with or without 2 μg per well of RGD as blocking agent at 37 °C for 0.5, 1 and 2h. The cells were washed 3 times with PBS and lysed in 0.5 mL of 1.0 M NaOH and tested with a γ-counter (Perkin-Elmer model 1470). Cell uptake of **^{68}Ga-CHS2** was expressed as the percentage of added radioactive dose (%AD). Experiments were performed twice with triplicate wells.

Cell viability. *In vitro* cytotoxicity of **CHS2** and ^{nat}Ga-CHS2 were determined in U87MG cells by the MTT assay. U87MG cells were incubated on 96-well plate in DMEM medium containing 10% FBS and 1% penicillin/streptomycin at 37 °C in 5% CO_2 humidified atmosphere for 24 h and 0.5×10^4 cells were seeded per well. Cells were then cultured in the medium supplemented with indicated doses of different **CHS2** for 24 h. The final concentrations of **CHS2** and ^{nat}Ga-CHS2 in the culture medium were fixed at 0.5, 1, 2 and 4 mM in the experiment. Addition of 10 μL of MTT (0.5 mg/mL) solution to each well and incubation for 3 h at 37 °C was followed to produce formazan crystals. Then, the supernatant was removed and the products were lysed with 200 μL of DMSO. The absorbance value was recorded at 590 nm using a microplate reader. The absorbance of the untreated cells was used as a control and its absorbance was as the reference value for calculating 100% cellular viability.

***In vivo* PET imaging of tumors.** The U87MG xenograft-bearing mice were injected with 3.5-7.5 MBq of **^{68}Ga-CHS2** *via* the tail vein (n = 3 for each group). At the indicated times after injection (0.5, 1, and 2 h), the mice were anesthetized with isoflurane (5% for induction and 2% for maintenance in 100% O_2) and subjected to PET/CT imaging. Blocking studies were performed *via* tail vein co-injection of RGD (n=3).

***In vivo* NIR-II fluorescence imaging.** For tumor imaging, animals were mounted on the imaging stage in the prone position beneath the laser. NIR-II fluorescence images were collected using a

NIR-II imaging system which was purchased from Suzhou NIR-Optics Technologies CO., Ltd. The excitation light was provided by an 808-nm diode laser. The laser power density was 82 mW/cm^2 during imaging and 105 mW/cm^2 during photostability studies.

***Ex vivo* biodistribution analysis.** 2 h after injection of **^{68}Ga-CHS2** for PET imaging or 60 h after injection of **CHS2** for NIR-II imaging, U87MG xenograft mice (n = 3 per group) were sacrificed, the major organs were collected. The radioactivity was measured using a γ-counter and the NIR-II fluorescent signal of each organ was then measured by the NIR-II imaging system.

Histological Analysis. To assess the histology of para-cancerous tissues and tumor tissues, mice were sacrificed after imaging-guided surgery. Para-cancerous tissues and tumors were taken from the tumor-bearing mice and fixed with 10 % buffered formalin. Tissue samples were stained with hematoxylin and eosin (H&E) and subsequently imaged using a digital microscope at different magnifications.

Chemical synthesis and characterization

Synthesis of 1

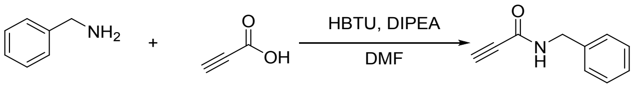

1

Synthesis of 1: A solution of benzylamine (107mg, 1mmol), in dry DMF (3 mL) was bubbled with argon for 10 min. Propiolic acid (70mg, 1 mmol), HBTU (380mg, 1 mmol) and DIPEA (2 mmol) were added to the mixture and stirred at room temperature for 4h. After complete conversion of the starting material, the resulting solution was filtered through a bed of silica gel. After the filtrate was evaporated in vacuo, the residue was subjected to column chromatography (silica gel, petroleum:EtOAc=20:1) to afford 124mg compound **1** as a white solid, Yield 78%. ^{1}H NMR (400 MHz, $CDCl_3$) δ 7.38-7.28 (m, 5H), 6.17 (br, 1H), 4.50 (d, J = 4.0 Hz, 2H), 2.81 (br, 1H). ^{13}C NMR (101 MHz, $CDCl_3$) δ 152.1, 136.9, 129.1, 128.8, 127.9, 127.9, 127.2, 73.7, 43.9. ESI-MS Calcd for: $C_{10}H_{10}NO^+$ ($[M+H]^+$): 160.0, found: 160.1.

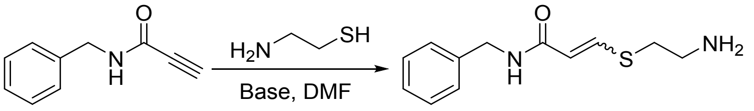

1 **1a (cis/trans mixture)**

Synthesis of 1a: To ananhydrous DMF (0.4 mL) solution of compound 1 (15.9 mg, 0.1 mmol) was added Cysteamine (7.7 mg, 0.1 mmol) and with or without base (0.1 eq). The mixture was

stirred at room temperature for 0.5h. Water was added to the reaction, after filtration, the mixture was extracted 3 times with diethyl ether. The organic phase was washed with water and brine then dried (Na_2SO_4). The solvent was removed under vacuum to obtain a white solid (52-85%). The cis/trans mixture was further isolated by HPLC to obtain the cis isomer as major products. The cis isomer was checked by ^{1}HNMR and ^{13}CNMR. ^{1}H NMR (400 MHz, MeOD) δ 7.37 – 7.09 (m, 5H), 6.96 (d, *J* = 10.1 Hz, 1H), 5.97 (d, *J* = 10.0 Hz, 1H), 4.37 (s, 2H), 2.84 – 2.81 (m, 2H), 2.80 – 2.77 (m, 2H).^{13}C NMR (101 MHz, MeOD) δ 168.7, 146.6, 140.1, 129.6, 128.7, 128.3, 116.9, 43.9, 42.9, 39.4.ESI-MS Calcd for: $C_{12}H_{17}N_2OS^+$ ($[M+H]^+$): 237.1, found: 237.1.

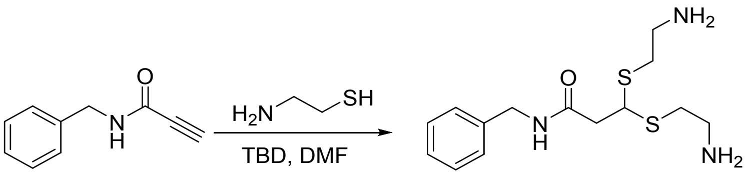

1 **1b**

Synthesis of 1b: To ananhydrous DMF(0.4 mL) solution of compound 1(15.9 mg, 0.1 mmol) was added Cysteamine (30.8 mg, 0.4mmol) and with TBD (0.2eq). The mixture was stirred at room temperature for 1h. Water was added to the reaction, after filtration, the mixture was extracted 3 times with diethyl ether. The organic phase was washed with water and brine then dried (Na_2SO_4). The solvent was removed under vacuum to obtain a white solid (20.9 mg, 67%). ^{1}H NMR (400 MHz, MeOD) δ 7.34-7.25 (m, 5H), 4.40 (s, 2H), 3.21-3.18 (m, 4H), 3.05-2.89 (m, 5H), 2.83 (d, *J* = 7.6 Hz, 2H). ^{13}C NMR (101 MHz, MeOD) δ 128.2, 127.2, 126.9, 42.9, 42,2, 38.7, 27.6. ESI-MS Calcd for: $C_{14}H_{24}N_3OS_2^+$ ($[M+H]^+$): 314.1, found: 314.1.

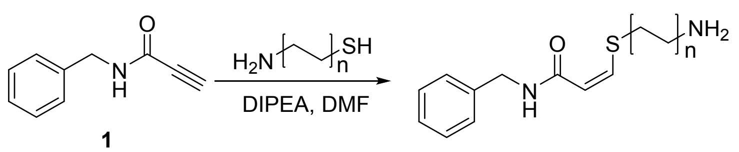

1 **1c-1d**

Major product

n = 3 60 min, 74%

n = 5 60 min, 68%

Synthesis of 6-aminohexane-1-thiol: The synthesis of 6-aminohexane-1-thiol was according to previous paper[2,3]. The crude was purified by silica gel to obtain the desire product as yellow oil .^{1}H NMR (400 MHz, MeOD) δ 2.70-2.66 (m, 2H), 2.53 (t, *J* = 7.1 Hz, 2H), 1.67-1.60 (m,

2H),1.56-1.36 (m, 8H). ^{13}C NMR (101 MHz, MeOD) δ 40.9, 33.8, 31.8, 27.8, 26.0, 23.5. ESI-MS Calcd for: $C_6H_{16}NS^+$ ($[M+H]^+$):134.1, found: 134.1.

Synthesis of 10-aminodecane-1-thiol: The synthesis of 10-aminodecane-1-thiol was according to previous paper[2,3]. The crude was purified by silica gel to obtain the desire product as yellow solid . ^{1}H NMR (400 MHz, $CDCl_3$) δ 2.70-2.61 (m, 2H), 2.57-2.43 (m, 2H), 1.65-1.51 (m, 2H), 1.43-1.21 (m, 16H). ^{13}C NMR (101 MHz, $CDCl_3$) δ 42.2, 34.0, 33.7, 29.5, 29.4, 29.0, 28.3, 26.8, 24.6. ESI-MS Calcd for: $C_{10}H_{24}NS^+$ ($[M+H]^+$):190.1, found: 190.3.

Synthesis of 1c: To an anhydrous DMF(0.4 mL) solution of compound 1 (15.9 mg, 0.1 mmol) was added 6-aminohexane-1-thiol (13.3 mg, 0.1 mmol) and with DIEPA (0.1 eq). The mixture was stirred at room temperature for 1h. Water was added to the reaction, after filtration, the mixture was extracted 3 times with diethyl ether. The organic phase was washed with water and brine then dried (Na_2SO_4). The solvent was removed under vacuum to obtain a light yellow solid (21.6 mg, 74%). The cis/trans mixture was further isolated by HPLC to obtain the cis isomer as major products.^{1}H NMR (400 MHz, MeOD) δ 7.31-7.21 (m, 5H), 7.00 (d, J = 12.0 Hz, 1H), 5.93 (d, J = 12.0 Hz, 1H), 4.38 (s, 2H), 2.92 (t,J = 8.0 Hz, 2H), 2.75 (t,J = 8.0 Hz, 2H), 1.71-1.61 (m, 5H), 1.51-1.40 (m, 5H).^{13}C NMR (101 MHz, MeOD) δ 167.4, 145.7, 138.7, 128.1, 127.1, 126.7, 114.5, 42.4, 39.2, 35.2, 29.7, 27.4, 27.0, 25.5. ESI-MS Calcd for: $C_{16}H_{25}N_2OS^+$ ($[M+H]^+$): 293.2, found: 293.1.

Synthesis of 1d: To an anhydrous DMF (0.4 mL) solution of compound**1** (15.9 mg, 0.1 mmol) was added 10-aminodecane-1-thiol (20.3 mg, 0.1 mmol) and with DIEPA (0.1 eq). The mixture was stirred at room temperature for 1h. Water was added to the reaction, after filtration, the mixture was extracted 3 times with diethyl ether. The organic phase was washed with water and brine then dried (Na_2SO_4). The solvent was removed under vacuum to obtain a light yellow oil (24.5 mg, 68%). The cis/trans mixture was further purified by HPLC to obtain the cis isomer as major products.^{1}H NMR (400 MHz, MeOD) δ 7.31-7.21 (m, 5H), 6.98 (d, J = 8.0 Hz, 1H), 5.91 (d, J = 8.0 Hz, 1H), 4.38 (s, 2H), 2.91 (t,J = 8.0 Hz, 2H), 2.74 (t,J = 8.0 Hz, 2H), 1.68-1.6158 (m, 5H), 1.33 (br, 13H).^{13}C NMR (101 MHz, MeOD) δ 145.8, 128.1, 127.1, 126.7, 114.5, 42.4, 39.4, 35.5, 29.9, 28.9, 28.8, 28.7, 28.6, 27.9, 27.1, 25.9. ESI-MS Calcd for: $C_{20}H_{33}N_2OS^+$ ($[M+H]^+$): 349.2, found: 349.3.

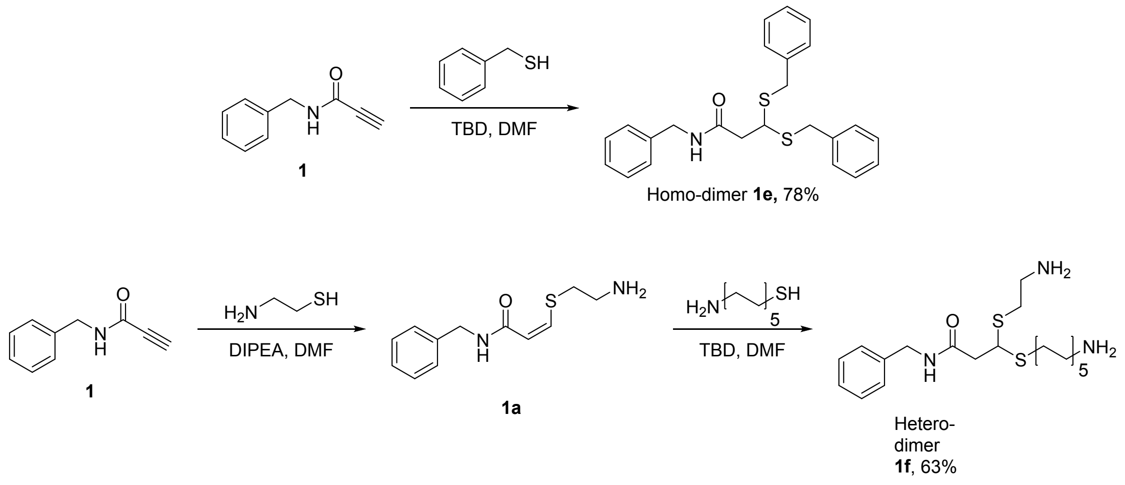

Synthesis of 1e: To an anhydrous DMF (0.4 mL) solution of compound **1** (15.9 mg, 0.1 mmol) was added benzyl mercaptan (49.6 mg, 0.4 mmol) and with TBD (0.2 eq). The mixture was stirred at room temperature for 1h. Water was added to the reaction, after filtration, the mixture was extracted 3 times with diethyl ether. The organic phase was washed with water and brine then dried (Na_2SO_4). The solvent was removed under vacuum to obtain a light yellow solid (31.8 mg, 78%). ^1H NMR (400 MHz, $CDCl_3$) δ 7.34-7.21 (m, 15H), 4.34 (d, J = 8.0 Hz, 2H), 4.07 (t, J = 8.0 Hz, 1H), 3.80 (d, J = 4.0 Hz, 4H), 2.54 (d,J = 4.0 Hz, 2H). ^{13}C NMR (101 MHz, Methanol) δ 169.0, 138.0, 137.8, 129.0, 128.6, 127.8, 127.5, 127.2, 46.9, 43.7, 43.3, 35.2. ESI-MS Calcd for: $C_{24}H_{26}NOS_2^+$ ($[M+H]^+$): 408.1, found: 408.5.

Synthesis of 1f: To an anhydrous DMF(0.4 mL) solution of compound 1a (23.6 mg, 0.1 mmol) was added 10-aminodecane-1-thiol (40.6 mg, 0.2mmol) and with TBD (0.2 eq). The mixture was stirred at room temperature for 1h. Water was added to the reaction, after filtration, the mixture was extracted 3 times with diethyl ether. The organic phase was washed with water and brine then dried (Na_2SO_4). The solvent was removed under vacuum to obtain a light yellow oil (26.8 mg, 63%). ^1H NMR (400 MHz, MeOD) δ 7.35-7.20 (m, 5H), 4.44-4.28 (m, 4H), 3.18 (t, J = 8.0 Hz, 2H), 2.91-1.31 (m, 11H), 1.67-1.57 (m,5H), 1.57-1.31 (br,12H). ^{13}C NMR (101 MHz, MeOD) δ 170.4, 138.2, 128.1, 127.2, 126.8, 42.8, 42.6, 39.4, 38.7, 29.9, 29.1, 29.0, 28.9, 28.9, 28.8, 28.6, 27.8, 27.2, 26.0. ESI-MS Calcd for: $C_{22}H_{40}N_3OS_2^+$ ($[M+H]^+$): 426.2, found: 427.3.

Synthesis of JMV594: Peptide JMV594 ((D)Phe-Gln-Trp-Ala-Val-Gly-His-Sta-Leu-NH_2) was synthesized on Tentagel S RAM resin using traditional Fmoc solid-phase peptide chemistry. After

deprotection and cleavage from the resin using 93% TFA, 5% Tips, and 2% H_2O for 2 h, the peptide was precipitated in cold Et_2O and washed with Et_2O three times. The dried peptide was purified by prep-HPLC. MS Calcd for: $C_{55}H_{81}N_{14}O_{11}^+$ ($[M+H]^+$): 1113.6, found: m/z 1113.7

Synthesis of AE105: Peptide AE105 (Ac-Lys-Gly-Asp-Cha-Phe-(D)Ser-(D)Arg-Tyr-Leu-Trp-Ser -NH2) was synthesized on Tentagel S RAM resin using traditional Fmoc solid-phase peptide chemistry. After deprotection and cleavage from the resin using 93% TFA, 5% Tips, and 2% H_2O for 2 h, the peptide was precipitated in cold Et_2O and washed with Et_2O three times. The dried peptide was purified by prep-HPLC. MS Calcd for: $C_{70}H_{102}N_{17}O_{17}^+$ ($[M+H]^+$): 1452.8, found: m/z 1452.3

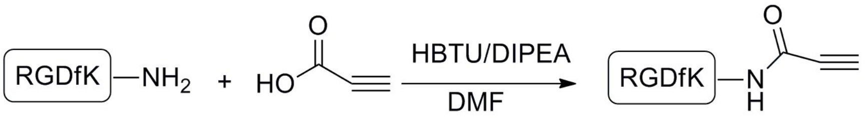

2a

Synthesis of 2a: A solution of c(RGDfK) (6.38 mg, 0.01mmol), in dry DMF (100 μL) was bubbled with argon for 10 min. propiolic acid (1mg, 0.015 mmol), HBTU (3.80 mg, 0.01 mmol) and DIPEA (0.02 mmol) were added to the mixture and stirred at room temperature for 4h. The mixture was diluted with water and then purified by HPLC to afford 4.64 mg compound **2a** as a white solid, Yield 72%. ESI-MS, Calcd for: $C_{30}H_{42}N_9O_8^+$ ($[M+H]^+$): 656.3, found: 656.5.

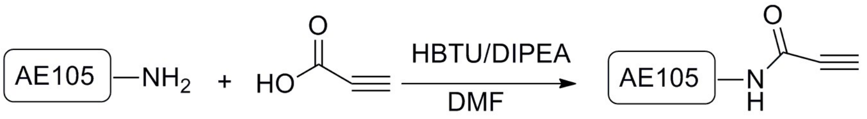

2b

Synthesis of 2b: A solution of AE105 (7.26mg, 0.005mmol), in dry DMF (100 μL) was bubbled with argon for 10 min. Propiolic acid (0.5mg, 0.007mmol), HBTU (1.90 mg, 0.005mmol) and DIPEA (0.01mmol) were added to the mixture and stirred at room temperature for 4h. The mixture was diluted with water and then purified by HPLC to afford 5.11mg compound **2b** as a white solid, Yield 68%. ESI-MS Calcd for: $C_{73}H_{102}N_{17}O_{18}^+$ ($[M+H]^+$): 1504.7, found: 1504.6.

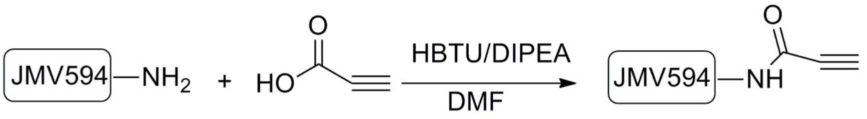

2c

Synthesis of 2c: A solution of JMV594 (5.56 mg, 0.005mmol), in dry DMF (100 μL) was bubbled with argon for 10 min. Propiolic acid (0.5mg, 0.007 mmol), HBTU (1.90 mg, 0.005 mmol) and

DIPEA (0.01 mmol) were added to the mixture and stirred at room temperature for 4h. The mixture was diluted with water and then purified by HPLC to afford 4.36mg compound **2c** as a white solid, Yield 75%. ESI-MS Calcd for: $C_{58}H_{81}N_{14}O_{12}^{+}$ ($[M+H]^{+}$): 1165.6, found: 1165.5.

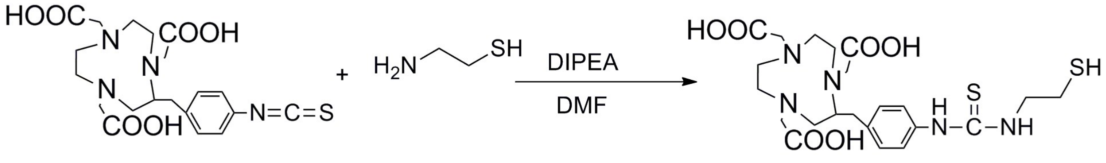

Y1

Synthesis of Y1: A solution of NOTA-NCS (4.50mg, 0.01mmol), in dry DMF (100 μL) was bubbled with argon for 10 min. Cysteamine (0.77 mg, 0.0071mmol), and DIPEA (0.02mmol) were added to the mixture and stirred at room temperature for 2h. The mixture was diluted with water and then purified by HPLC to afford4.36 mg compound **Y1** as a white solid, Yield 75%. ESI-MS, Calcd for: $C_{22}H_{34}N_5O_6S_2^{+}$ ($[M+H]^{+}$): 528.2, found: 528.4.

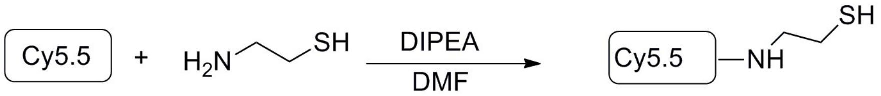

Y2

Synthesis of Y2: A solution of Cy5.5-NHS ester (2.148 mg, 0.003mmol), in dry DMF (100 μL) was bubbled with argon for 10 min. Cysteamine (0.69 mg, 0.009 mmol), and DIPEA (0.01 mmol) were added to the mixture and stirred at room temperature for 2h. The mixture was diluted with water and then purified by HPLC to afford 1.23 mg compound **Y2** as a blue powder, Yield 65%. ESI-MS Calcd for: $C_{42}H_{48}N_3OS^{+}$ ($[M+H]^{+}$): 642.4, found: 642.7.

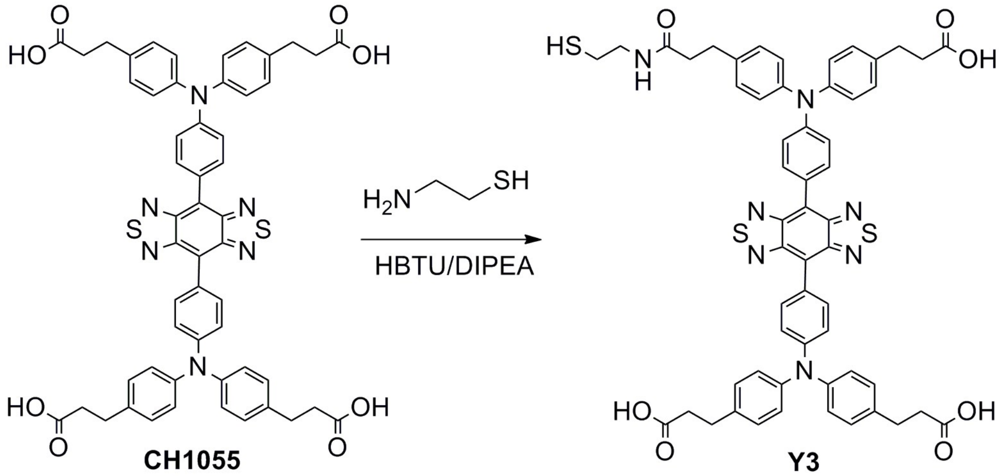

CH1055 **Y3**

Synthesis of Y3: A solution of CH1055 (2.90 mg, 0.003mmol), in dry DMF (100 μL) was bubbled with argon for 10 min. Cysteamine (0.23 mg, 0.003 mmol), HBTU (1.14 mg, 0.003 mmol) and

DIPEA (0.01 mmol) were added to the mixture and stirred at room temperature for 4h. The mixture was diluted with water and then purified by HPLC to afford 1.23 mg compound **Y3** as a green powder, Yield 65%. ESI-MS Calcd for: $C_{56}H_{49}N_7O_7S_3^+$ ($[M+H]^+$): 1028.3, found: 1028.1.

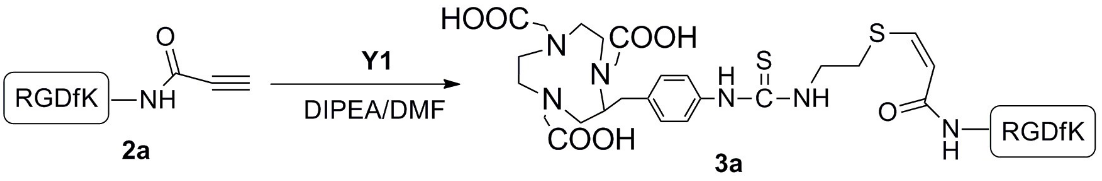

Synthesis of 3a: A solution of **2a** (0.65mg, 0.001mmol), in dry DMF (30μL) was bubbled with argon for 10 min. **Y1** (0.53mg, 0.001mmol) and DIPEA (0.1 eq) were added to the mixture and stirred at room temperature for 1.5h. The mixture was diluted with water and then purified by HPLC to afford 0.68mg compound **3a** as a white powder, Yield 58%. ESI-MS, Calcd for: $C_{52}H_{75}N_{14}O_{14}S_2^+$ ($[M+H]^+$): 1183.5, found: 1183.1.

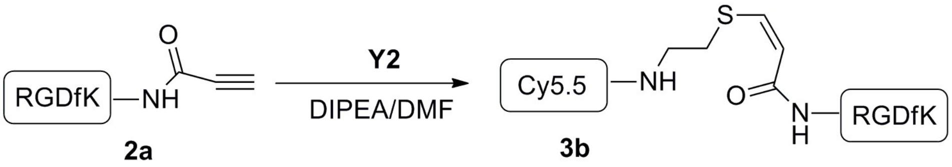

Synthesis of 3b: A solution of **2a** (0.65 mg, 0.001mmol), in dry DMF (30 μL) was bubbled with argon for 10 min. **Y2** (0.64mg, 0.001 mmol) and DIPEA (0.1 eq) were added to the mixture and stirred at room temperature for 1.5h. The mixture was diluted with water and then purified by HPLC to afford 0.78 mg compound **3b** as a blue powder, Yield 60%. ESI-MS, Calcd for: $C_{72}H_{89}N_{12}O_9S^+$ ($[M+H]^+$): 1297.6, found: 1297.9.

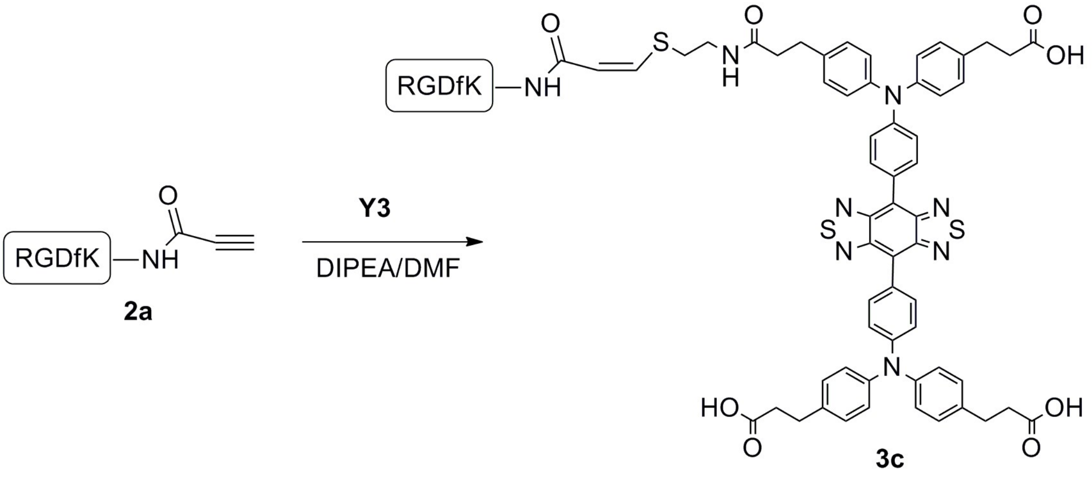

Synthesis of 3c: A solution of **2a** (0.65 mg, 0.001mmol), in dry DMF (30 μL) was bubbled with argon for 10 min. **Y3** (1.03mg, 0.001 mmol) and DIPEA (0.1 eq) were added to the mixture and

stirred at room temperature for 1.5h. The mixture was diluted with water and then purified by HPLC to afford 1.01mg compound **3c** as a green powder, Yield 60%. ESI-MS, Calcd for: $C_{86}H_{91}N_{16}O_{15}S_3^+$ ([M+H]$^+$): 1683.6, found: 1683.2.

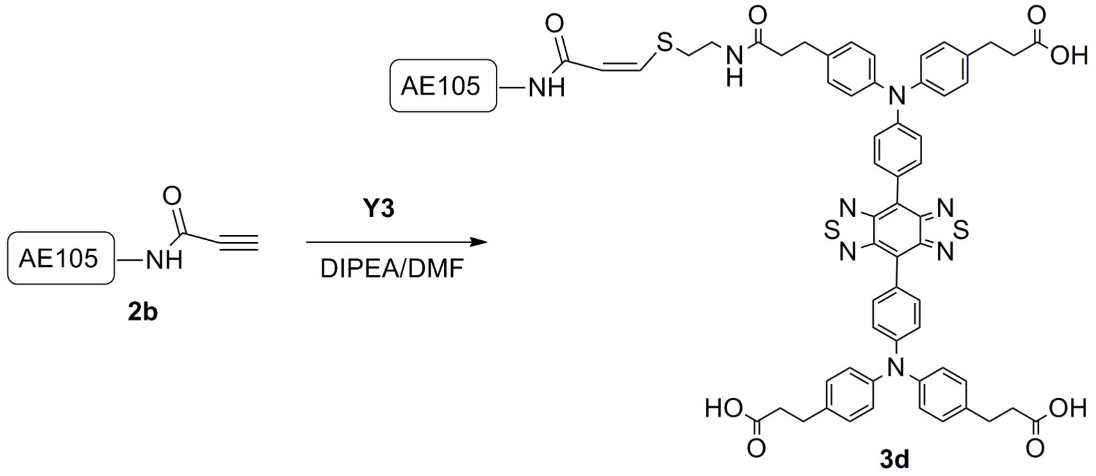

Synthesis of 3d: A solution of **2b** (1.50mg, 0.001mmol), in dry DMF (30 μL) was bubbled with argon for 10 min. **Y3** (1.03 mg, 0.001 mmol) and DIPEA (0.1 eq) were added to the mixture and stirred at room temperature for 1.5h. The mixture was diluted with water and then purified by HPLC to afford 1.51 mg compound **3d** as a green powder, Yield 61%. ESI-MS, Calcd for: $C_{129}H_{151}N_{24}O_{25}S_3^+$ ([M+H]$^+$): 2532.0, found: 2532.4.

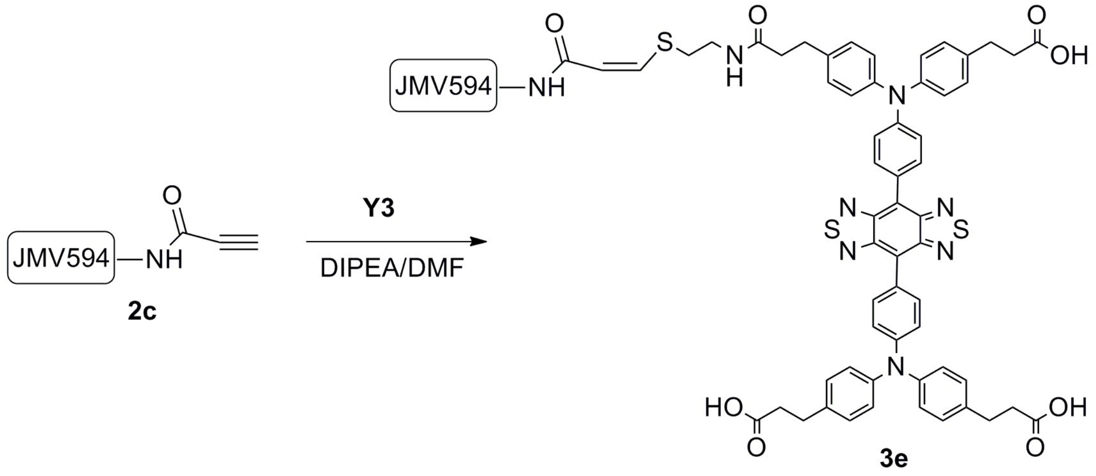

Synthesis of 3e: A solution of **2c** (1.16mg, 0.001mmol), in dry DMF (30 μL) was bubbled with argon for 10 min. **Y3** (1.03 mg, 0.001 mmol) and DIPEA (0.1 eq) were added to the mixture and stirred at room temperature for 1.5 h. The mixture was diluted with water and then purified by HPLC to afford 1.44 mg compound **3e** as a green powder, Yield 60%. ESI-MS, Calcd for: $C_{114}H_{129}N_{21}O_{19}S_3^+$ ([M+H]$^+$): 2191.9, found: 2192.2.

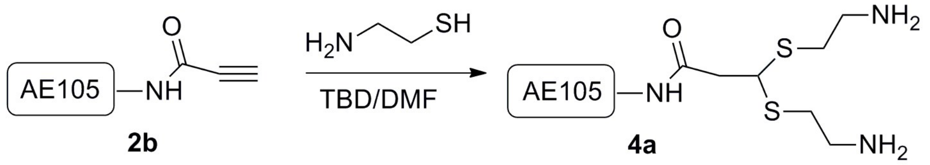

Synthesis of 4a: A solution of **2b** (1.50 mg, 0.001mmol), in dry DMF (30 μL) was bubbled with argon for 10 min. Cysteamine (0.31mg, 0.004mmol) and TBD (0.2eq) were added to the mixture and stirred at room temperature for 2h. The mixture was diluted with water and then purified by HPLC to afford 0.97mg compound **4a** as a white powder, Yield 58%. ESI-MS, Calcd for: $C_{77}H_{116}N_{19}O_{18}S_2^+$ ($[M+H]^+$): 1658.8, found: 1660.0.

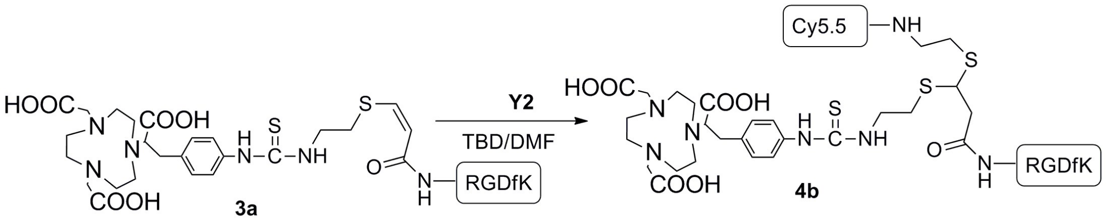

Synthesis of 4b: A solution of **3a** (1.18 mg, 0.001mmol), in dry DMF (40 μL) was bubbled with argon for 10 min. **Y2** (1.28 mg, 0.002 mmol) and TBD (0.2 eq) were added to the mixture and stirred at room temperature for 2 h. The mixture was diluted with water and then purified by HPLC to afford 0.86mg compound **4b** as a blue powder, Yield 47%. ESI-MS, Calcd for: $C_{94}H_{122}N_{17}O_{15}S_3^+$ ($[M+H]^+$): 1824.8, found: 1824.5.

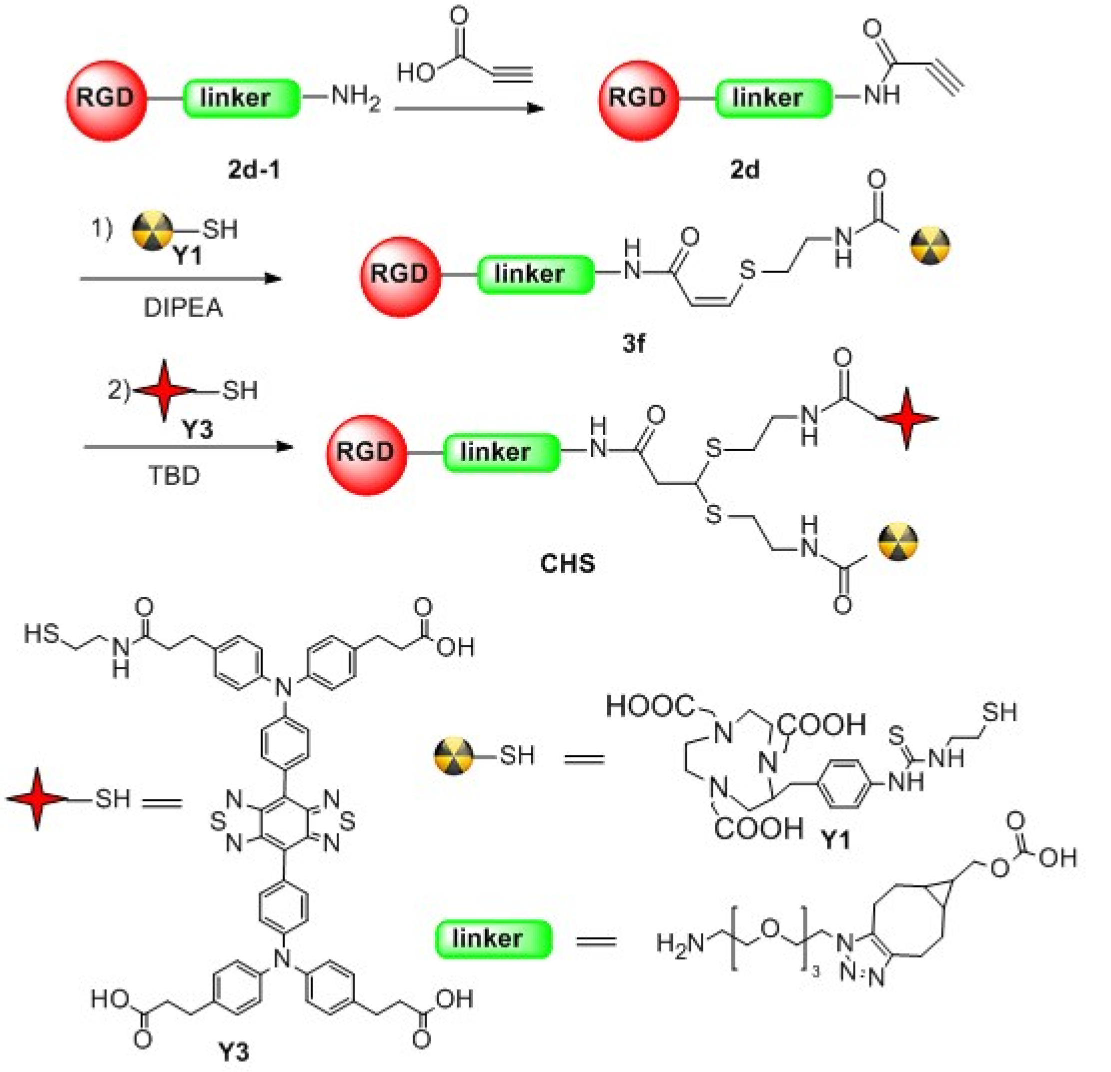

Synthesis of CHS

The compound **2d-1** was obtained by two steps coupling and followed the previous paper.[4] The crude product was purified by HPLC and checked by ESI-MS. MS Calcd for: $C_{78}H_{118}N_{23}O_{23}^{+}$ ([M+H]$^{+}$): 1744.8, found: ESI-MS: m/z 1744.8.

To a solution of compound **2d-1** (3.50 mg, 0.002mmol), propiolic acid(0.22 mg, 0.003mmol) and HBTU (0.72 mg, 0.002mmol) in DMF at room temperature. The reaction mixture was stirred at this temperature for 4 h. The crude product was purified by HPLC. Lyophilization of the purified material gave 2.44 mg (68%) of **2d**. MS Calcd for: $C_{81}H_{118}N_{23}O_{24}^{+}$ ([M+H]$^{+}$): 1795.8, found: ESI-MS: m/z 1795.4.

A solution of **2d** (1.80 mg, 0.001mmol), in dry DMF (30 μL) was bubbled with argon for 10 min. **Y1** (0.53 mg, 0.001 mmol) and DIPEA (0.1 eq) were added to the mixture and stirred at room temperature for 1.5 h. The mixture was diluted with water and then purified by HPLC to afford

1.27mg compound **3f** as a white powder, Yield 55%., MS Calcd for: $C_{103}H_{151}N_{28}O_{30}S_2^+$ ([M+H]$^+$): 2323.0, found: MALDI-TOF-MS, 2324.3.

A solution of **3f** (1.16 mg, 0.0005mmol), in dry DMF (20 μL) was bubbled with argon for 10 min. **Y3** (1.27 mg, 0.001 mmol) and TBD (0.2 eq) were added to the mixture and stirred at room temperature for 2 h. The mixture was diluted with water and then purified by HPLC to afford 0.73mg compound **CHS** as a green powder, Yield 42%. MS Calcd for: $C_{159}H_{200}N_{35}O_{37}S_5^+$ ([M+H]$^+$): 3351.3, found: MALDI-TOF-MS, 3350.2.

NMR Spectra

^{1}H and ^{13}CNMR for 1

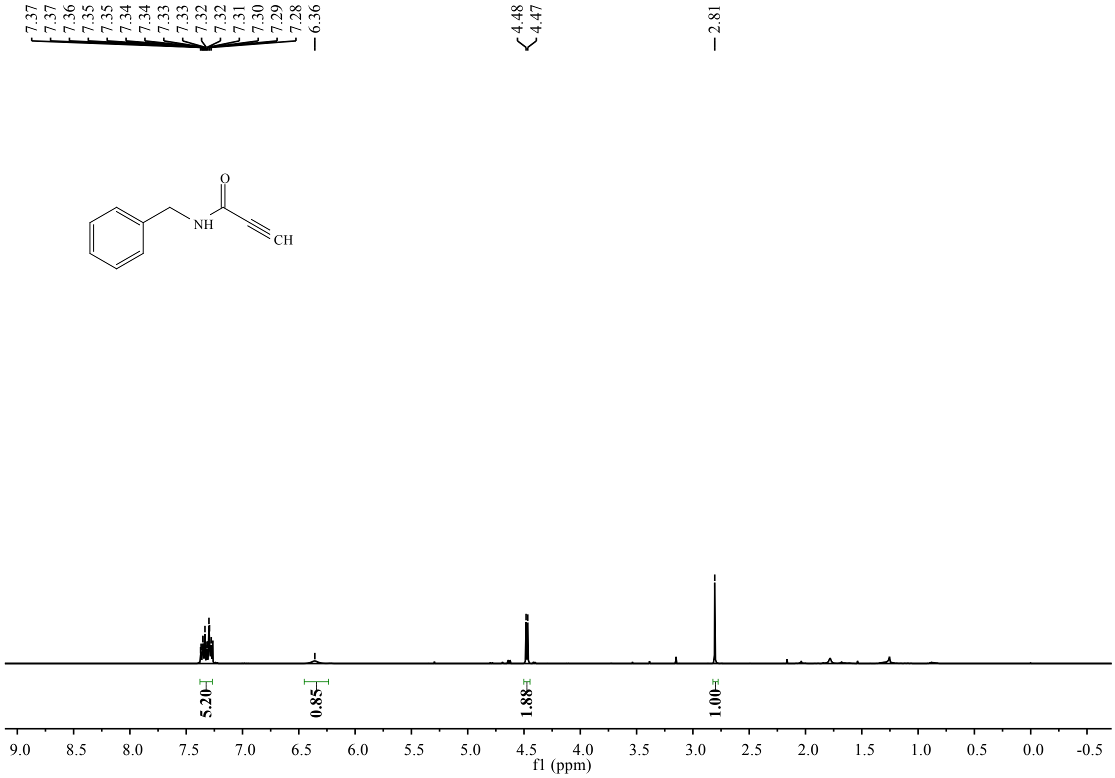

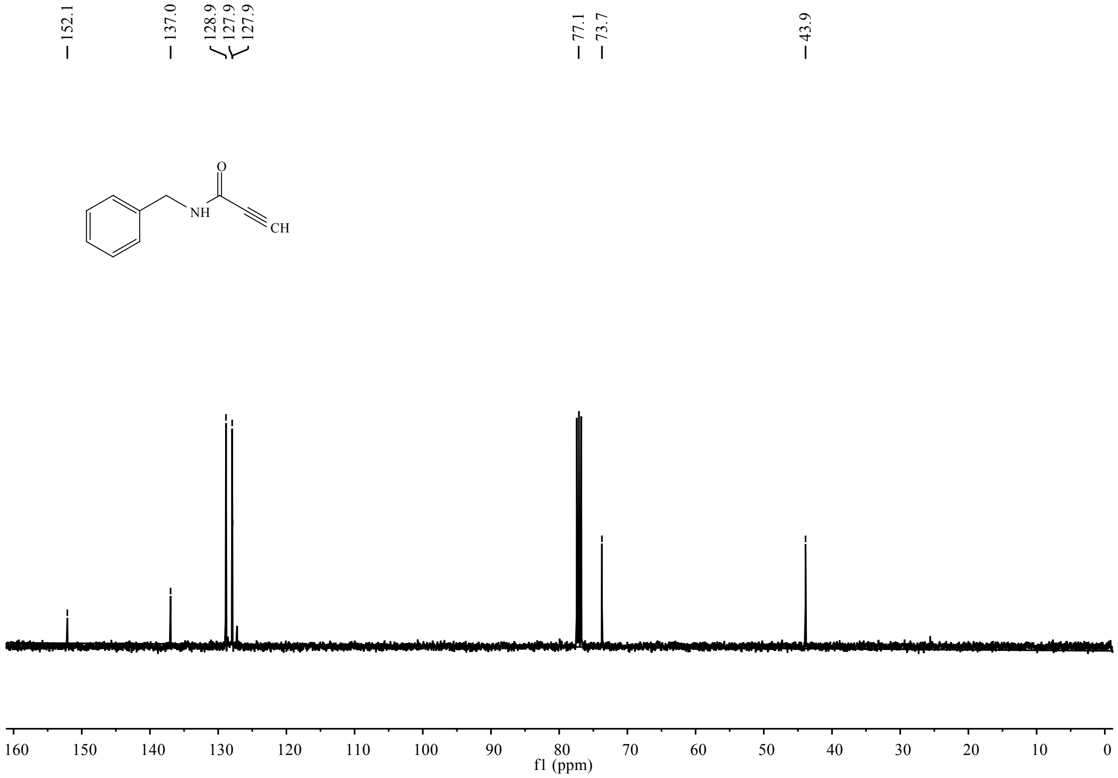

^{1}H and ^{13}CNMR for 1c

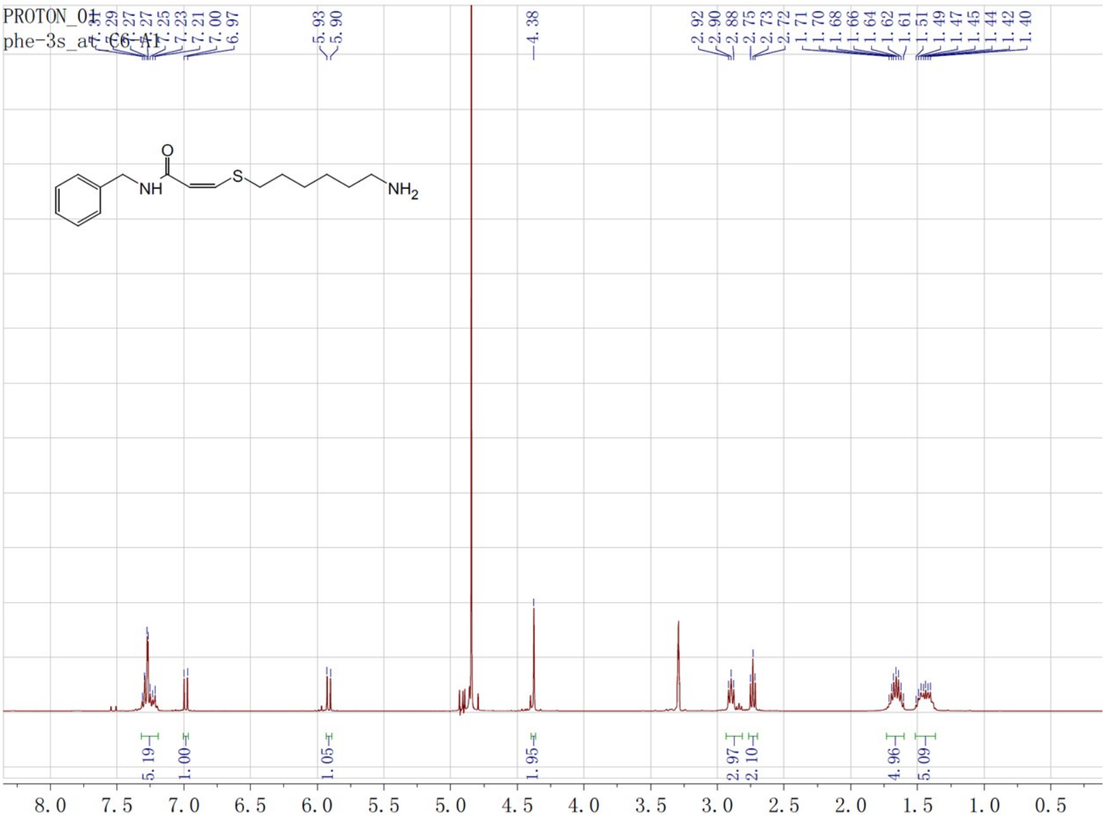

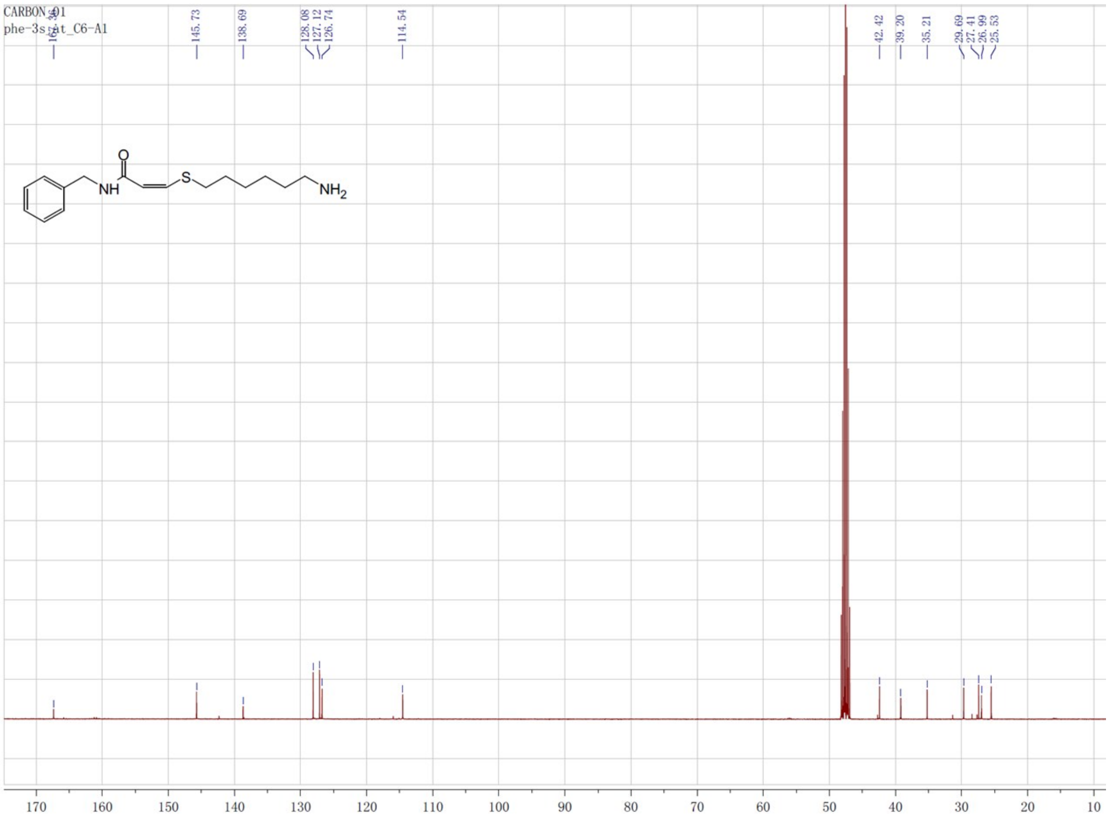

^{1}H and ^{13}CNMR for 1d

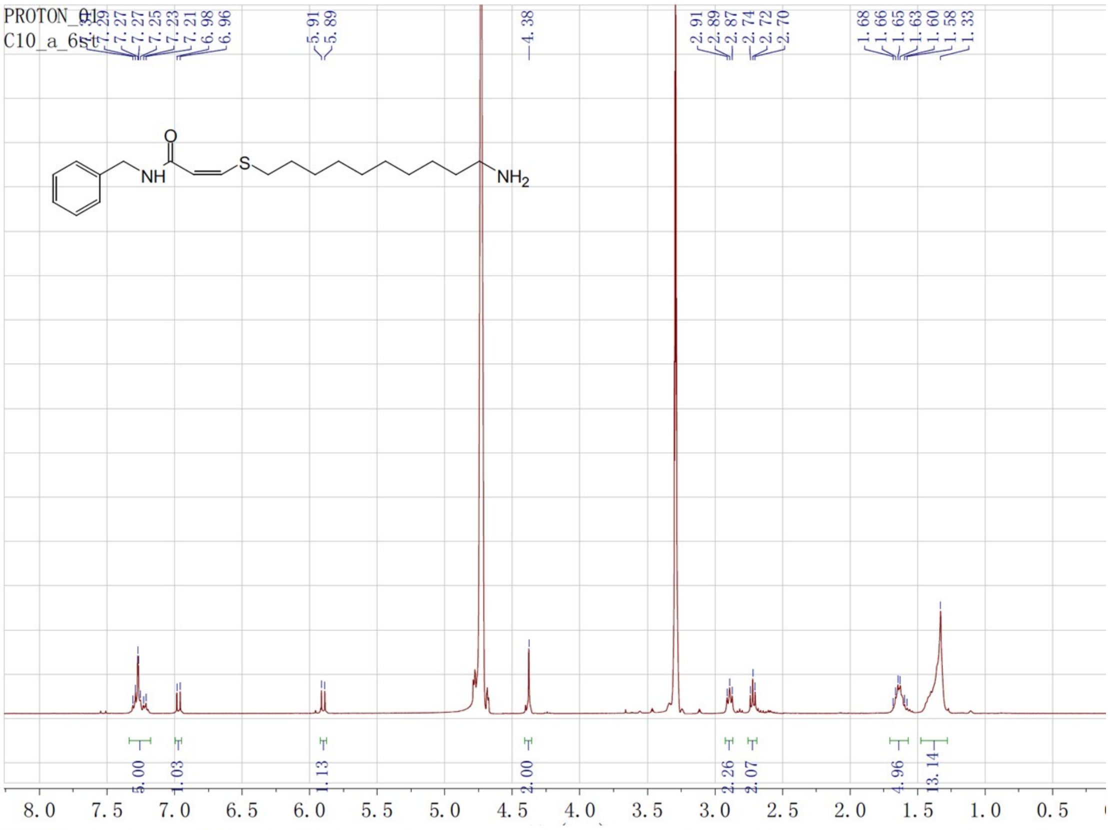

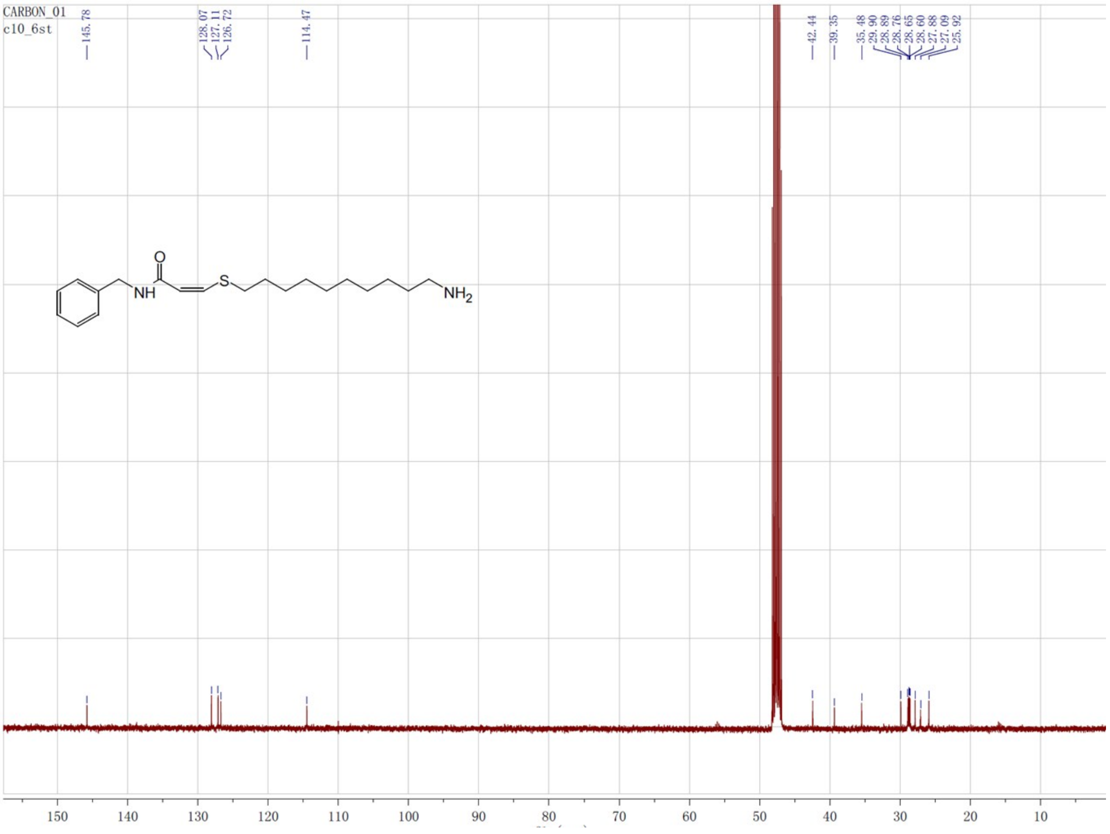

^{1}H and ^{13}CNMR for 4

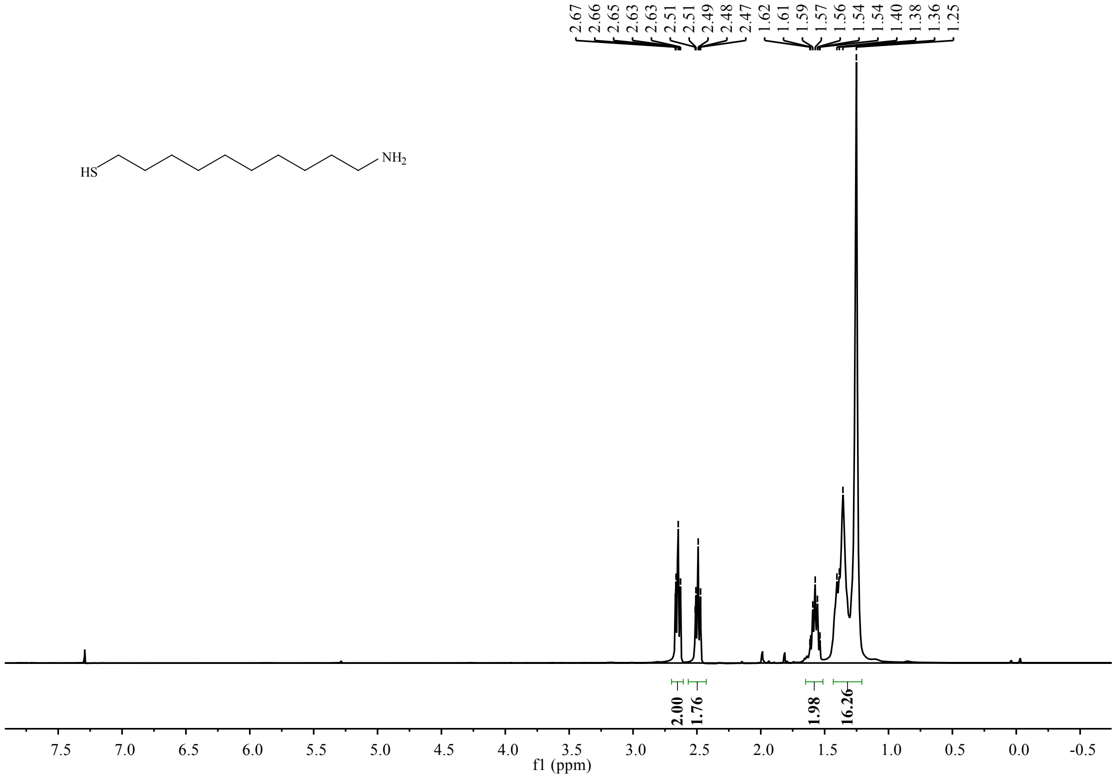

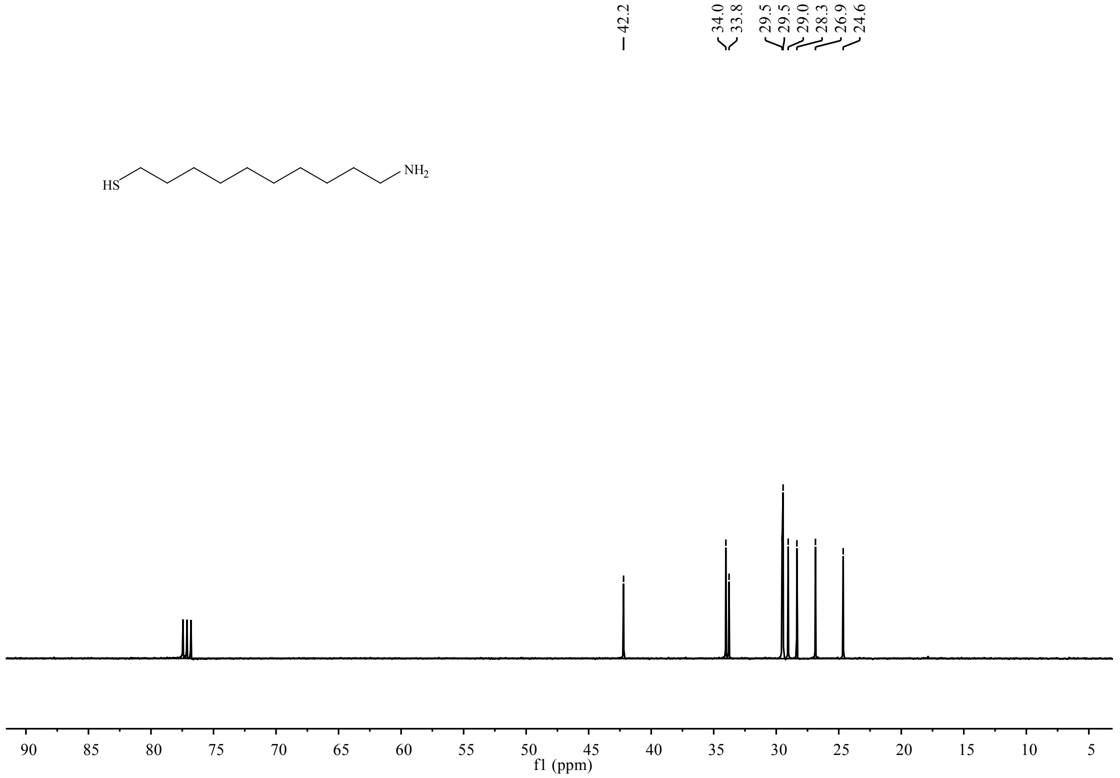

^{1}H and ^{13}CNMR for 5

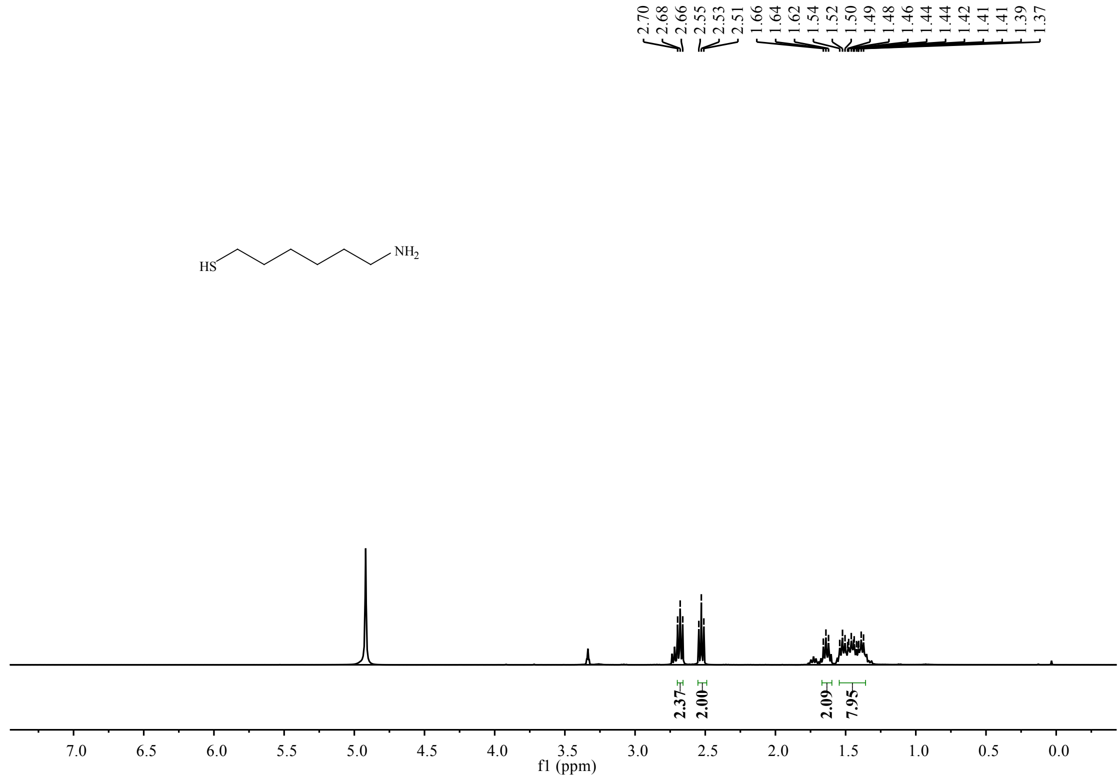

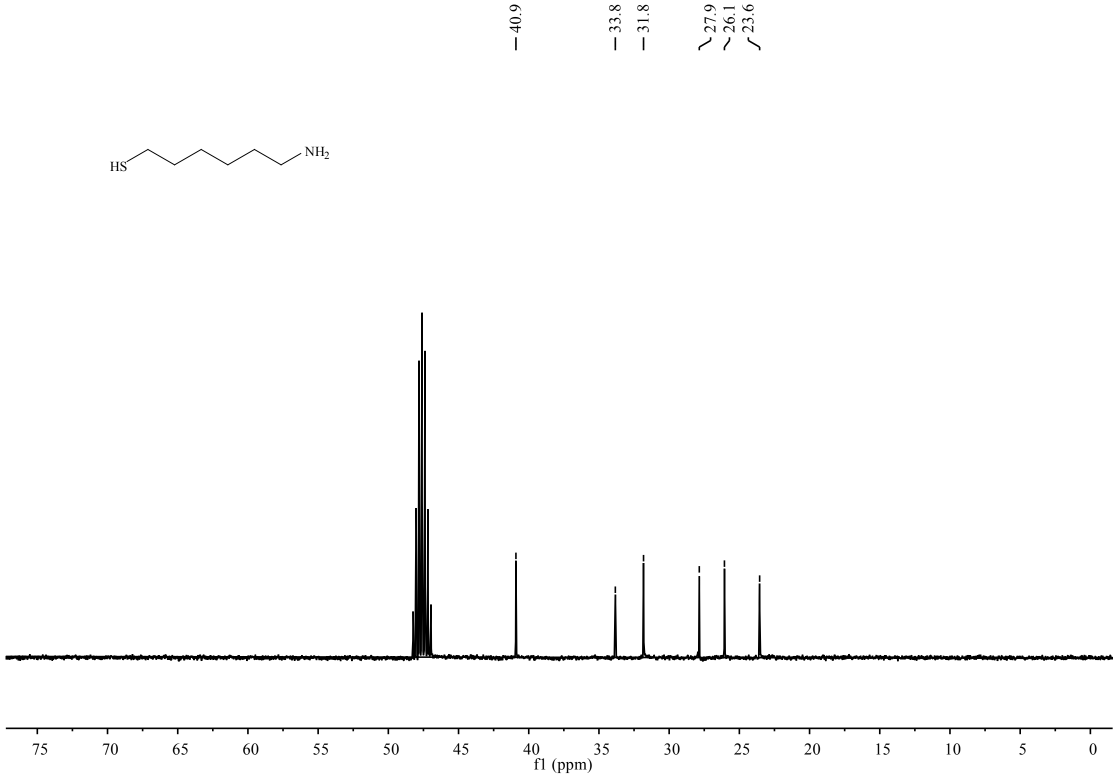

References

[1] Sun Y, Ding M, Zeng X, *et al*. Novel bright-emission small-molecule NIR-II fluorophores for in vivo tumor imaging and image-guided surgery. *Chem. Sci.*, **2017**, 8: 3489-3493.

[2]Lefèvre X, Moggia F, Segut O, *et al*. Influence of molecular organization on the electrical characteristics of π-conjugated self-assembled monolayers. *J. Phys. Chem. C*, **2015**, *119(10)*, 5703-5713.

[3] Hu K, Qi Y, Zhao J, *et al*. Synthesis and biological evaluation of sulforaphane derivatives as potential antitumor agents. *Eur. J. Med. Chem.*, **2013**, *64*, 529-539.

[4] Sun Y, *et al*. Novel benzo-bis(1,2,5-thiadiazole) fluorophpres for in vivo NIR-II imaging of cancer. *Chem. Sci.*, **2016**, *7*, 6203-6207.
